# Modelling the regional potential for reaching carbon neutrality in Finland: Sustainable forestry, energy use and biodiversity protection

**DOI:** 10.1007/s13280-023-01860-1

**Published:** 2023-08-10

**Authors:** Martin Forsius, Maria Holmberg, Virpi Junttila, Heini Kujala, Torsti Schulz, Ville-Veikko Paunu, Mikko Savolahti, Francesco Minunno, Anu Akujärvi, Jaana Bäck, Juha Grönroos, Risto K. Heikkinen, Niko Karvosenoja, Annikki Mäkelä, Ninni Mikkonen, Minna Pekkonen, Katri Rankinen, Raimo Virkkala

**Affiliations:** 1 Finnish Environment Institute SYKE, Latokartanonkaari 11, 00790 Helsinki, Finland; 2 Finnish Natural History Museum, University of Helsinki, (Pohjoinen Rautatiekatu 13), P.O. Box 17, 00014 Helsinki, Finland; 3 Institute for Atmospheric and Earth System Research (INAR) & Faculty of Agriculture and Forestry, University of Helsinki, (Latokartanonkaari 7), P.O. Box 27, 00014 Helsinki, Finland

**Keywords:** Biodiversity, Carbon, Emissions, Forests, Greenhouse gases, Scenarios

## Abstract

The EU aims at reaching carbon neutrality by 2050 and Finland by 2035. We integrated results of three spatially distributed model systems (FRES, PREBAS, Zonation) to evaluate the potential to reach this goal at both national and regional scale in Finland, by simultaneously considering protection targets of the EU biodiversity (BD) strategy. Modelling of both anthropogenic emissions and forestry measures were carried out, and forested areas important for BD protection were identified based on spatial prioritization. We used scenarios until 2050 based on mitigation measures of the national climate and energy strategy, forestry policies and predicted climate change, and evaluated how implementation of these scenarios would affect greenhouse gas fluxes, carbon storages, and the possibility to reach the carbon neutrality target. Potential new forested areas for BD protection according to the EU 10% protection target provided a significant carbon storage (426–452 TgC) and sequestration potential (− 12 to − 17.5 TgCO_2_eq a^−1^) by 2050, indicating complementarity of emission mitigation and conservation measures. The results of the study can be utilized for integrating climate and BD policies, accounting of ecosystem services for climate regulation, and delimitation of areas for conservation.

**Supplementary Information:**

The online version contains supplementary material available at 10.1007/s13280-023-01860-1.

## Introduction

Reliable assessment of anthropogenic carbon dioxide (CO_2_) and greenhouse gas (GHG) emissions and their redistribution among the atmosphere, ocean, and terrestrial biosphere in a changing climate is important to better understand the global carbon (C) cycle, support the development of climate policies, and project future climate change (Friedlingstein et al. 2020). Net negative GHG emissions are needed to comply with the targets of the Paris climate agreement (Rogelj et al. 2019; IPCC 2022). The projected economic mitigation potential of options in the global land sector between 2020 and 2050 is 8–14 GtCO_2_eq a^−1^ (IPCC 2022). Consequently, estimation of national/regional-scale C budgets for different land use classes and their future developments is important for the design of different management and mitigation measures, and for the evaluation potential feedbacks to the climate system (Mäkipää et al. 2015; Morecroft et al. 2019; Forsius et al. 2021; Holmberg et al. 2021).

Forests play a key role in these global and regional climate change mitigation efforts (IPCC 2022). The EU Regulation for the Land Use, Land Use Change and Forestry (LULUCF) sector (EU 2018) creates the legislative framework for emissions and removals from the land use sector. According to the recent provisional agreement on the update of the LULUCF regulation, the sector will have a new EU target of − 310 MtCO_2_eq for year 2030. Recently, many countries, institutions and companies have announced plans to reach “C neutrality,” “climate neutrality” or “net zero emissions,” where the general aim is to achieve a balance between sources and sinks of CO_2_ or GHGs by a target year. However, such plans are hard to compare because of varying assumptions, such as inclusion of different GHGs and target years, which may complicate international negotiations (Rogelj et al. 2021).

According to the recent update of the Finnish Climate Act, the country should be C neutral by the year 2035 and aiming for net negative GHG emissions thereafter. Total Finnish GHG emissions were 49.8 TgCO_2_eq in 2021 (Statistics Finland 2022) and the LULUCF sector was for the first time an emission source (0.9 TgCO_2_eq). During previous years, the LULUCF sector has been a sink varying from approximately 3 to 55% of the total annual emissions from other sectors during 1990 to 2020 (Statistics Finland 2022). In Finland, forests cover ca. 69% of the area. Thus, the forestry sector is totally dominating the LULUCF sink category. Variations in forest harvesting is the main reason causing the large deviations in the net C sink values, both in the past and in the projected future scenarios (Finnish Climate Change Panel 2021).

The challenges posed by climate change, land use and biodiversity (BD) loss are deeply interconnected problems, and detrimental effects of human actions on BD and ecosystem services (ESs) are observed across the globe/at the global scale (Diaz et al. 2019; Pörtner et al. 2021). Formal plans to increase BD protection and enhance ES have, therefore, been made at both international, e.g., the EU BD Strategy for 2030 (EU 2020), and national scales. The concept of ES accounting is also receiving increasing attention (Dasgupta 2021; UN 2021). The aim of ES accounting is to develop a comprehensive framework for organizing data about habitats and landscapes, measuring the ES, tracking changes in ecosystem assets, and linking this information to economic and other human activity. In these efforts, “climate regulation services” is one of the key ES under consideration (UN 2021). In a similar vein, and following the EU BD strategy, the national Finnish BD strategy includes several measures such as consideration of climate change impacts, enhancing the network of protected areas, accounting for the maintenance of BD and ES in land use planning, and halting the decline in forest species and habitats. Currently 31% of the threatened species live primarily in the forested ecosystems of the country (Hyvärinen et al. 2019). The Finnish Nature Panel has recently proposed that at least 10% of the remaining old-growth and valuable forest should be strictly protected in each administrative region in Finland, following the recommendation of the EU BD strategy. The portion of strictly protected areas is currently significantly higher in northern Finland than elsewhere (Kotiaho et al. 2021). This would have consequences also for forest harvesting and the C balances of these systems, and hence the national GHG budget.

Under these multiple targets, a key challenge is therefore to find a balance between anthropogenic GHG emissions, actual and potential future C sequestration/storage in the ecosystems (i.e., climate regulation services), BD conservation, use of land resources, and other environmental impacts. Spatially explicit dynamic modelling and optimization methods which can support sustainable resource management and explore potential win–win or trade-off situations regarding both climate change mitigation and conservation are therefore needed (Buotte et al. 2020; Reside et al. 2020; Forsius et al. 2021; Blattert et al. 2022).

The aims of this study were to: (i) develop a methodology for detailed spatial and dynamic evaluation of C and GHG processes in the context of C neutrality evaluations, with special focus on forested ecosystems and anthropogenic point and areal GHG emissions; (ii) evaluate the impact of forestry measures and quantitative BD protection targets on the C budgets and climate related ES indicators of forested ecosystems; (iii) assess the possibilities to reach the national C neutrality target within different time-frames, using scenarios for anthropogenic GHG emissions, forest harvesting, BD protection targets of forests, and climate change; (iv) aggregate key results to 18 administrative regions covering the country; and (v) provide a conceptual and integrated methodological framework and blueprint for similar studies in other countries and regions.

The forested ecosystems are given a special attention because of their large spatial cover, dominating role regarding C sequestration/storage, and importance for species protection in Finland. We synthesize the results of three different model systems into a coherent framework and show how this information can assist in developing integrated solutions for climate change mitigation, forest management and conservation planning. The study is a further development and extension of our previous work in this field (Forsius et al. 2021; Holmberg et al. 2021).

## Materials and methods

### Model and data descriptions

#### Forest growth and gas exchange model PREBAS

The PREBAS model was used to simulate forest growth, management and GHG balance in the study areas. Details of the PREBAS modelling and scenario derivation are given in the accompanying papers of Junttila et al. (2023) and Mäkelä et al. (2023) and are here only summarised. PREBAS is a C balance-based stand growth and gas exchange model (Minunno et al. 2019). Photosynthesis (GPP) and evapotranspiration are calculated using a light-use efficiency approach linked to soil moisture and driven by daily climate information and ambient CO_2_ concentration. GPP is allocated to mean-tree growth and respiration at an annual time step. For calculating NEE (net ecosystem exchange), PREBAS has been linked with the soil C model Yasso07 through annual litter inputs (Tuomi et al. 2009). For forests on drained peatland soils, soil respiration estimates were based on measured soil respiration, which includes both peat and litter decomposition (or accumulation) (Minkkinen et al. 2018). The model has been calibrated using information from Nordic eddy covariance eLTER/ICOS sites and Finnish growth experiments (Minunno et al. 2016, 2019). PREBAS requires information on the initial state of the simulated forest, and forest management actions, including clearcut, thinning, and regeneration. The initial state of forests in forest C balance simulations was determined based on MS-NFI (Multi Source-National Forest Inventory) maps, which describe the forest parameters as thematic maps across Finland at 16 × 16 m resolution. MS-NFI maps are developed by combining the information from NFI field measurements, satellite imagery and digital map data (Tomppo et al. 2008). To decrease computational load, similar forest areas were segmented, with a mean size of 3926 m^2^.

PREBAS was used to calculate NEE (gC m^−2^ a^−1^) for current and future climate conditions, and the amount of harvested biomass (gC m^−2^ a^−1^). The harvested amount for each simulation year was specified by realized removals statistics until 2021. Removals data defined the total amount of harvests in the study area. Total NEE was calculated as a sum of stand and soil fluxes, and it acquired positive values when the C flux from the decomposition of soil organic matter was larger than the assimilation of C into growing vegetation, and negative when the assimilation of C into growing vegetation was larger than the C flux from decomposing soil organic matter. The GHG balance and net emissions of forests were estimated with net biome exchange (NBE) calculated as: NBE = NEE + harvested biomass + CH_4_ and N_2_O flux from organic soils (gCO_2_eq m^−2^ a^−1^). The forest acts as a source of GHGs to the atmosphere when the net emissions are positive, and as a sink when the net emissions are negative. The soil C storage in drained peatlands was estimated according to Turunen and Valpola (2020). The C balance of drained peatlands was calculated using the PREBAS model for trees and ground vegetation and empirical coefficients for the soil. The empirical coefficients determined the emissions from the soil and are positive for nutrient-rich drained peatlands (source) and negative for nutrient-poor drained peatlands (small sink) (Ojanen et al. 2019).

Uncertainties of C flux and C storage related output variables were estimated based on direct Monte-Carlo simulations. Different sources of uncertainty were given as samples from input and parameter distributions for the individual simulations, and the resulting samples of the simulation outputs were used to estimate the uncertainty in given time points or time intervals (Junttila et al. 2023).

#### FRES anthropogenic emission model

Anthropogenic CO_2_, CH_4_ and N_2_O emissions in the study areas were estimated using the FRES (Finnish Regional Emission Scenario) model (Karvosenoja 2008; Karvosenoja et al. 2020) for emissions from combustion of fuels, industrial processes, and peat production. At the time of calculations, present-day emissions were based on the latest national fuel use data (Statistics Finland 2020b) and represent the year 2019. Emissions from agriculture and waste management were calculated with the ALas (Regional Calculation) model (Lounasheimo et al. 2020), with spatial downscaling methods of the FRES model. The emissions are given separately for point sources on municipal level and area sources on 250 m × 250 m resolution. The point source emissions indicate the emissions from major industrial-sized facilities with known locations, calculated based on representative fuel mixes and annual operating hours for energy production plants and 3-year average emissions for industrial processes. For biogenic fuels, CO_2_ emissions were not included to avoid double-counting. Since the FRES model is used for spatial allocation of emissions calculated by both FRES and ALas models, estimates of anthropogenic emissions are subsequently referred to as FRES model results.

The FRES model uses several proxies to estimate the spatial distribution of the area source emissions (Paunu et al. 2013; Karvosenoja et al. 2020). The main data sources for the proxies were Digiroad for roads and traffic volumes, The National Buildings and Dwellings Register for Buildings data and CORINE2012 for land use data. The area sources were aggregated to six sectors: traffic exhaust (CO_2_), machinery and off-road (CO_2_), small scale wood combustion (CH_4_, N_2_O), other small-scale combustion (CO_2_), agriculture (CO_2_, CH_4_, N_2_O) and waste management (CH_4_, N_2_O). Agricultural emissions were estimated for enteric fermentation (CH_4_), manure management (CH_4_, N_2_O), agricultural soils (N_2_O), field burning of agricultural residues (CH_4_, N_2_O), liming (CO_2_) and urea application (CO_2_). Emissions from the waste sector were estimated for solid waste disposal (CH_4_), biological treatment of solid waste (CH_4_, N_2_O) and wastewater treatment (CH_4_, N_2_O). Emissions from peat production were calculated with the emission factors used in the national GHG inventory (Statistics Finland 2020a, b). For more information, see Holmberg et al. (2021).

The estimation of uncertainty of the emissions was based on source and GHG-level uncertainty intervals. The uncertainty intervals were results of activity data and emissions coefficient uncertainties (Statistics Finland 2020a, b; Holmberg et al. 2023). Quantitative uncertainty estimates are given only at country-level. The limitations and challenges of the uncertainty estimates are discussed below (“Limitations” section).

#### Calculation and mapping of present day GHG-fluxes and C storages for different land-use classes

The present-day GHG fluxes for all major land-use classes in Finland were calculated to quantify their relative importance. These calculations are described in detail in the accompanying paper of Holmberg et al. (2023). For each land cover type the average annual net emissions of one or several GHGs (CO_2_, CH_4_, N_2_O, depending on the key processes occurring in respective ecosystems) were calculated as the sum of sources (positive emissions) and sinks (negative emissions). The annual emissions represent the best estimates of current conditions, allowing for some variation in the time frames of the available data (see Holmberg et al. 2023). For forests, PREBAS model results and for artificial surfaces FRES model results were used. For lakes, rivers, mires and peat extraction sites and arable land annual vertical fluxes of GHGs between the atmosphere and surfaces were calculated with area-specific emission factors. The different gas fluxes to the atmosphere were converted to CO_2_eq to express the fluxes in comparable units. Spatial data sources included CORINE land use data, soil map, lake and river shorelines, national forest inventory data, and statistical data on anthropogenic activities. C storages for the different land-use classes and ecosystem compartments were estimated based on PREBAS modelling for the forested areas (this study) and published literature values for other classes.

#### Zonation model for spatial BD prioritization

We used the spatial prioritization tool Zonation (v.5.0) (Moilanen et al. 2005, 2022) to identify important forested areas for additional BD protection, according to the 10% strict protection target. As input data, Zonation uses spatial data on the distribution of different BD values: (i) the dead wood potential of forest stands, described in Mikkonen et al. (2018, 2020) and calculated separately for different forest site type and dominant tree species combinations (*n* = 20); (ii) distribution of old-growth forest stands likely to include valuable BD elements, delineated separately for three dominant tree species (Forsius et al. 2021) (*n* = 3); (iii) observation frequency of National Red List forest species (Forsius et al. 2021) (*n* = 1); and (iv) modelled nesting suitability for six forest-dwelling bird species indicative of forest stands with conservation value (Virkkala et al. 2022) (*n* = 6). All the source data for these different BD data layers were converted or resampled using R (R Core Team 2022) to a uniform 96 × 96 m grid covering the study area. All input data was weighted equally.

Zonation produces a hierarchal priority ranking of each spatial unit (here 96 × 96 m grid cells) based on feature data (maps of 30 BD features), ordering the cells from least to most important for conservation in a manner that maximizes the representation of all features in the top ranked grid cells. The number of values remaining for each input feature is tracked throughout the prioritization, so that all features are captured in the solution in a balanced manner (Moilanen et al. 2011, 2022; Kujala et al. 2023). Consequently, a set of top ranked priority areas together typically capture high value areas for all input features.

In the Zonation prioritization task, we accounted for past forestry actions (logging and management) at each pixel by penalizing locations where forest management and drainage of wet forests have changed tree volume and the naturalness of forest. The penalty values (a multiplier with values 0–1) were constructed separately for two groups of BD values, (i) dead wood potential on herb-rich soils; (ii) all other BD values and were based on the intensity and frequency of management and time since management actions. The prioritization focused on unprotected areas, but we also included information from protected areas, to identify those priority forests that best complement the current reserve network. The priority ranking was produced for the whole country and then used to identify areas for additional protection in each region. Effectively, in each region, areas for protection were identified by selecting grid cells with the highest rank value until the 10% protection target was met. Hence, although the targets were set and achieved regionally, the ranking guiding the selection of sites for protection was based on national priorities. The approach to use a regional 10% protection target was based on the recommendation of the Finnish Nature Panel (Kotiaho et al. 2021).

### Scenarios

The forest harvesting scenarios and their assumptions were based on the national climate and energy strategy (Koljonen et al. 2017, 2021) and forestry planning. The realised harvest levels of Finland were used as the target harvest levels in the simulations for the years 2015–2021, with a yearly average of total harvested volume approaching 80 Mm^3^. For the years 2022–2050, the harvest scenario BaseHarv describes a case where current management practises are assumed to continue also in the future. Target harvest level in MaxHarv was set to 1.2 × BaseHarv intensity level to describe very intense harvests. The LowHarv scenario describes moderate harvesting levels, and here 0.6 × BaseHarv intensity was assumed. A scenario with no harvesting after 2021 was also used (NoHarv). Harvests were allocated only to the productive forest land; no cuttings were performed in protection areas and poorly productive forest land. The scenarios and related forest modelling have been described in detail in Junttila et al. (2023) and Mäkelä et al. (2023). The PREBAS scenario results for the different regions were aggregated using GIS-techniques.

In addition to assuming continuation of current climate conditions, PREBAS was also run with a climate change scenario obtained as results of a sample of global climate models downscaled for Finland, assuming a stabilization scenario for a future pathway of radiative forcing (RCP4.5). RCP4.5 is a moderate scenario in which GHG emissions peak around 2040 and then decline. The resulting climate variables had been down-scaled to a 0.2° × 0.1° longitude–latitude grid (Holmberg et al. 2019; Junttila et al. 2023).

Two scenarios for future energy use have previously been prepared in two projects (called HIISI) to support the updates of national climate and energy strategy and the medium-term climate change policy plan. These were used as input to the FRES model. The scenarios were WEM (With Existing Measures): policies that had been decided by the end of 2019, and WAM (With Additional Measures): measures that help to meet additional goals of (i) C neutrality by 2035, and (ii) reduction of GHG emissions by 90% between 1990 and 2050. Assumptions of these scenarios are described in Koljonen et al. (2021, 2022). The scenarios presented in Koljonen et al. (2021, 2022) are subsequently called HIISI scenarios. In distinction, WEM and WAM scenarios subsequently refer to those calculated with the FRES model, where HIISI data is used to re-calculate and spatially allocate the national GHG emissions.

Fuel use input data of the WEM and WAM scenarios were received separately (A. Lehtilä, personal communication, March 21, 2022). Due to the aggregation of received HIISI data, distinction between all oil and gas types or the exact allocation of fuel use to all sectors and subsectors in the FRES model was not possible. Only country-level data for fuel use was provided in the scenarios. Outside of a few power/industrial plants that are being built or declared to be closed, we did not make assumptions on the type and location of plants that are being used in the future. We distributed the fuels uses of the WEM and WAM scenarios to the existing database of plants in the FRES model. The WAM scenario includes assumptions on large implementation of CCS (Carbon Capture and Storage) and BECCS (Bioenergy Carbon Capture and Storage) techniques which would reduce the anthropogenic GHG emissions by a total of about 9 TgCO_2_eq in 2050. We did not have information on the regional distribution of this CCS/BECCS implementation, and therefore we considered results of the WAM scenario only on the national level after 2030.

For the agricultural and waste sectors, the ALas model presents GHG emissions by municipality in each historical year. HIISI scenarios included the development of GHG emissions from those sectors in 2030 and 2050 on country-level. The same relative changes in country-level emissions from present-day have been used for all municipalities in the WEM and WAM scenarios.

### Integration of model results and spatial modelling

**Fig. 1:**
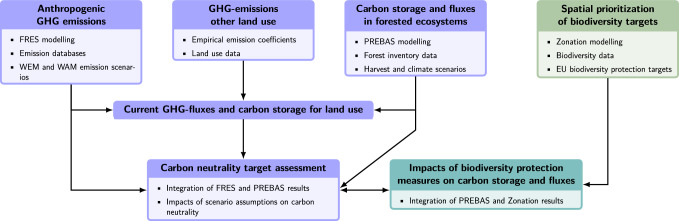
Framework and flow of data for modelling and evaluation of the study

Our integrated model framework connects different databases, anthropogenic emission, forest harvesting and climate scenarios, FRES, PREBAS and Zonation model results, and facilitates the flow of data between different compartments (Fig. 1). The FRES model results were used both to estimate the current anthropogenic GHG emissions and for assessing the future potential for reaching C neutrality. Similarly, PREBAS results were used for these two tasks, and in addition to estimate the change in C fluxes and storages in the areas for BD-protection. Spatial prioritization with Zonation was used to find the most important, as regards BD values, additional forested areas to reach the 10% target for BD protection (EU-target).

**Fig. 2:**
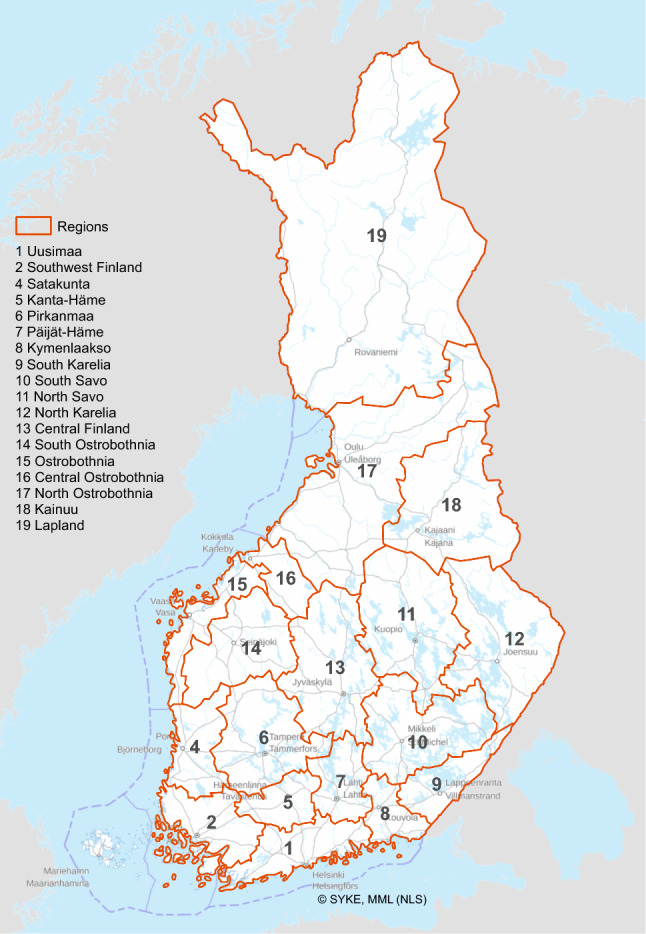
Location of Finnish administrative regions used in the spatial assessments of the study

All modelling was done at spatial (segment/grid) scale and then aggregated to each of the 18 administrative regions of mainland Finland (Fig. 2) and to national scale. These administrative regions are governed by councils, which serve as forums of cooperation for the municipalities of a region. Main tasks of the regions include regional planning and development of enterprise. In addition, there are 15 Centres for Economic Development, Transport, and the Environment (ELY-centres), which are responsible for the local administration of forestry, agriculture, and entrepreneurial affairs. They are each responsible for one or more of the 18 regions and thus potential regional end-users of the results of this study.

### Calculation of climate regulation services

Climate regulation services for the forested areas were calculated according to the SEEA (System of Environmental Economic Accounts) definition (EU 2021a): “Global climate regulation, defined as the ecosystem contributions to reducing concentrations of GHGs in the atmosphere through the removal (net sequestration) of carbon from the atmosphere and the retention (storage) of carbon in ecosystems. The contributions shall be reported in terms of tons of net sequestration of carbon and tons of organic carbon stored in terrestrial ecosystems including above ground and below ground in the first 0.3 m of the soil (including in peatlands).” These climate regulation service values for the forested areas were calculated with the PREBAS model, assuming the different forest harvesting scenarios (see above). The net C sequestration value was assumed to equal NBE, and the C storage value was calculated as the sum of above ground and soil C storage. The values were calculated for the whole country and the 18 administrative regions (Fig. 2).

## Results

### National scale estimates

#### Relative importance of current GHG emissions, sinks and C storages of different land-use classes and ecosystem compartments

We estimated the relative importance of present GHG emissions, sinks and C storages of different land-use classes and ecosystem compartments in mainland Finland based on the FRES and PREBAS model results and published values from the literature (Table 1). Anthropogenic emissions from artificial surfaces were at present clearly the largest source of GHG emissions (45.7 TgCO_2_eq a^−1^) and the forested areas (forest land and poorly productive forest land on mineral soils and drained peatlands) were the largest sink (− 89.3 TgCO_2_eq a^−1^) in Finland. However, large harvesting removals reduce the sink of the forested areas, and therefore the net emissions were estimated to be − 27.7 TgCO_2_eq a^−1^ (Table 1). Of the estimated harvesting emissions (61.6 TgCO_2_eq a^−1^), emissions from forest soils of CH_4_ and N_2_O were 0.4 and 1.4 TgCO_2_eq a^−1^, respectively (Holmberg et al. 2023). The values for the forested areas were estimated as averages of years 2017–2025 to account for variabilities in climatic conditions and forest harvesting. The C storages were the largest in undrained mires (2365 TgC) and peat and mineral soils of forested areas. The C storages in trees and ground vegetation biomass (924 TgC) and lake sediments were also significant (Table 1).

**Table 1:** Estimated current greenhouse gas (GHG) emissions and sinks (TgCO_2_eq a^−1^) and carbon (C) storages (TgC) according to land use classes and ecosystem compartments in mainland Finland. The values for GHG emissions and sinks are aggregated from Holmberg et al. (2023). Positive values indicate emissions and negative values sinks. Emissions from forests are due to harvesting removals and decomposition and respiration processes. The values for the forests were calculated as averages for several years to account for variability in climate conditions and harvesting removals. C storages (TgC) in different ecosystem compartments are estimated based on this study and published values from other studies. See text for details

Land use	Area (km^2^)	Area (%)	Emissions (TgCO_2_eq a^−1^)	Sinks (TgCO_2_eq a^−1^)	Net emission (TgCO_2_eq a^−1^)	Carbon storage (TgC)
Forests	211 130	69	61.6	− 89.3	− 27.7	924^1^
						2617^2^
						2358–2696^3^
Arable land	22 537	**7**	12.2		12.2	151–223^4^
						300^5^
Surface waters	33 896	11	13.4		13.4	600–630^6^
Wetland	31 534	10	14.3	− 3.9	10.4	2365^7^
Artificial surfaces	7973	3	45.7		45.7	
**Total**	**307 070**	**100**	**147.2**	** − 93.2**	**54.0**	

Bold indicate that these are totals

^1^Trees + ground vegetation biomass, this study

^2^Forest mineral and peat soil, this study

^3^Forestry drained peat soil (Turunen and Valpola 2020)

^4^Agricultural peat soils (area 2500 km^2^) (Turunen and Valpola 2020)

^5^Arable mineral land (Heikkinen 2016)

^6^Lake sediments (Kortelainen et al. 2004)

^7^Undrained mires (peat) (Turunen and Valpola 2020)

#### Impacts of forestry and anthropogenic emission scenarios on aggregated GHG emissions and sinks

The impact of forest harvesting scenarios and the RCP4.5 climate scenario on aggregated future GHG emissions and sinks of the forested areas were estimated using PREBAS. The results are shown in Table 2 for current climate conditions and the RCP4.5 climate scenario, respectively. The forest harvesting intensity scenarios were based on assumptions in the national climate and energy strategy and forestry policies and give a broad range in the total annual harvest removal quotas. Results for forests are shown as averages for several years to reduce the annual variability caused by changes in climate conditions. The results clearly indicate the substantial impact of the different harvesting removals on the net GHG emissions. Assuming continuation of current climate, the net GHG emissions (NBE) ranged from average values of − 34.6 TgCO_2_eq a^−1^ (Low scenario) to 25.0 TgCO_2_eq a^−1^ (MaxHarv scenario) for years 2026–2033 (Table 2). The rather intensive management assumed in the MaxHarv scenario would thus turn the forests into a large GHG emission source. The PREBAS results indicate an increase in NEE when the climatic conditions change according to scenario RCP4.5 (Table 2). Under the RCP4.5 climate projections for 2034–2050, NBE ranged between − 79.6 TgCO_2_eq a^−1^ (LowHarv) and − 6.2 TgCO_2_eq a^−1^ (MaxHarv).

**Table 2:** Impact of forest harvesting intensity and climate change scenarios (PREBAS model, current climate conditions and RCP4.5 scenario) and anthropogenic emission scenarios (FRES model) on aggregated GHG emissions and sinks (TgCO_2_eq a^−1^) in Finland. For the forest harvesting intensity scenarios (BaseHarv, LowHarv, MaxHarv), results for the different time periods and climate scenarios are shown (top row = current climate conditions; lower row = RCP4.5 scenario). For forests, emissions are assumed to equal harvested biomass and sinks NEE (net ecosystem exchange). The net emissions of forested areas (NBE) were calculated by adding NEE estimates to the amount of harvested biomass and estimating CH_4_ and N_2_O flux from organic soils. Positive values indicate emissions and negative values sinks. Results for forests are shown as averages for several years to reduce the annual variability caused by changes in climate conditions. The present situation refers to average result for years 2017–2025. FRES model results for the WEM (with existing measures) and WAM scenarios (with additional measures) for the anthropogenic emissions are shown. These scenarios are based on assumptions of the national climate and energy strategy. The best available estimates of uncertainty (95%) are shown for NEE, NBE, and WEM and WAM scenarios. See text for details

Scenario	Period	FRES emissions (95%) (TgCO_2_eq a^−1^)		Harvested volume (Mm^3^ a^−1^)	Harvested biomass (TgCO_2_eq a^−1^)	NEE (95%) (TgCO_2_eq a^−1^)		NBE (95%) (TgCO_2_eq a^−1^)	
BaseHarv	2017–2025			81.5 81.5	59.7 60.0	− 87.8 − 102	(− 101.0, − 70.4) (− 121.9, − 81.4)	− 28.0 − 42	(− 43.7, − 10.4) (− 61.9, − 21.7)
BaseHarv	2026–2033			85.1 85.1	63.6 63.7	− 60.4 − 91.5	(− 80.6, − 40.9) (− 112.2, − 68.5)	3.1 − 27.8	(− 16.4, 22.87) (− 46.5, − 5.1)
BaseHarv	2034–2050			81.3 83.5	61.1 62.4	− 45.3 − 88.1	(− 63.1, − 25.7) (− 113.8, − 60.8)	15.9 − 25.8	(− 0.0, 34.2) (− 51.4, − 1.2)
LowHarv	2017–2025			67.0 67.0	49.0 49.2	− 89.7 − 104.1	(− 104.7, − 72.4) (− 124, − 83.8)	− 40.8 − 54.9	(− 55.8, − 23.1) (− 75, − 36.5)
LowHarv	2026–2033			51.2 51.2	37.2 37.4	− 71.9 − 106	(− 90.2, − 57.5) (− 121.4, − 86.1)	− 34.6 − 68.6	(− 52.1, − 19.4) (− 84, − 49.4)
LowHarv	2034–2050			50.9 50.9	36.3 36.4	− 67.7 − 115.9	(− 80.2, − 52.6) (− 137.8, − 93.5)	− 31.4 − 79.6	(− 44.0, − 15.7) (− 102.8, − 57.9)
MaxHarv	2017–2025			88.7 88.7	65.0 65.3	− 85.2 − 99.1	(− 98.9, − 68.0) (− 119, − 79.1)	− 20.1 − 33.8	(− 35.9, − 2.0) (− 53.8, − 13.9)
MaxHarv	2026–2033			102.0 102.1	76.4 76.6	− 50.9 − 80.7	(− 71.51, − 31.13) (− 101, − 58.5)	25.0 − 4.1	(5.0, 42.0) (− 21.4, 17.6)
MaxHarv	2034–2050			89.9 94.8	68.0 71.2	− 35.1 − 77.4	(− 51.95, − 15.72) (− 103.1, − 53.3)	33.6 − 6.2	(21.3, 46.3) (− 29.6, 16.5)
FRES cur	2019	54.1	(48.1, 59.4)						
WEM	2030	39.0	(34.9, 44.8)						
WEM	2050	22.9	(20.0, 28.4)						
WAM	2030	33.4	(29.0, 39.0)						
WAM	2050	8.6/17.6	(15.0, 22.3)						

Future GHG emissions from anthropogenic point and areal sources were estimated using the FRES model, assuming the WEM and WAM scenarios based on the national climate and energy strategy (Table 2). Large emission reductions can clearly be achieved by the year 2050, with national emissions ranging from an average of 22.9 (WEM scenario) to 8.6/17.6 TgCO_2_eq a^−1^ (WAM scenario). The WAM value of 8.6 TgCO_2_eq a^−1^ is based on large-scale CCS/BECCS implementation (Koljonen et al. 2022). The results also indicate that the formal national policy goal of C neutrality by the year 2035 requires a considerable net GHG sink of the forested areas (or the entire LULUCF sector), since the WEM and WAM scenarios still indicate a limited emission reduction potential from the anthropogenic sources (Table 2).

**Fig. 3:**
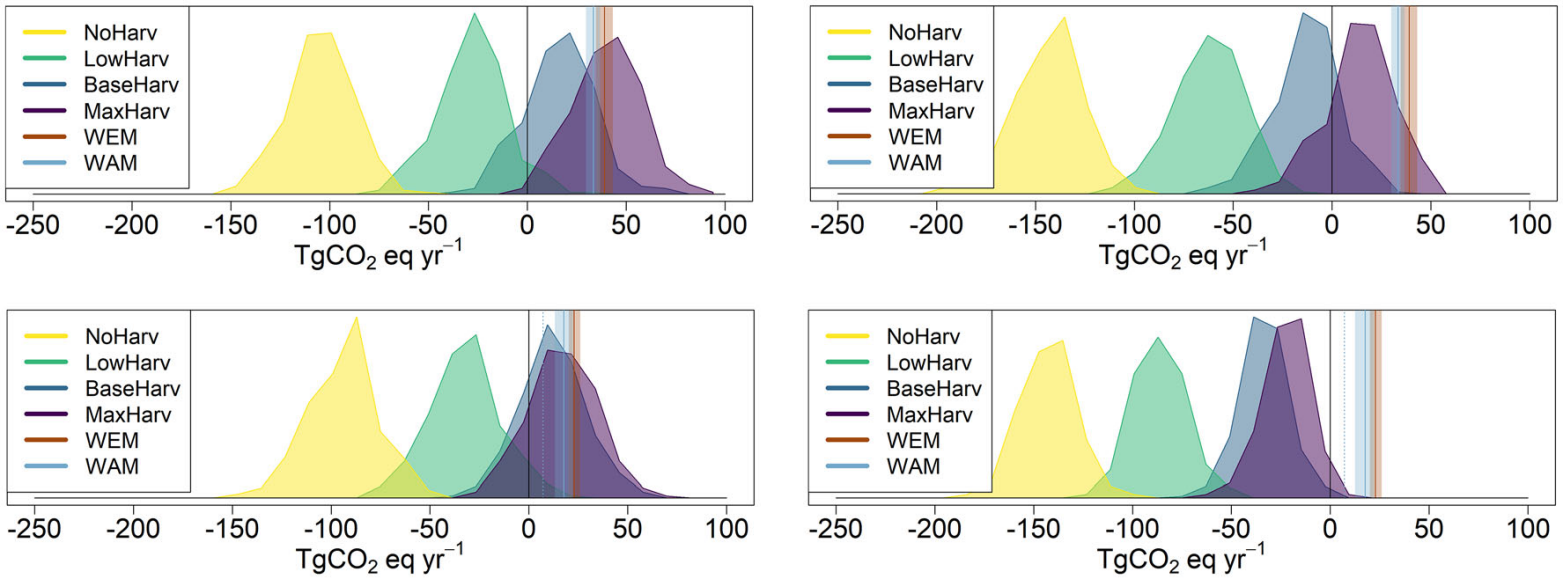
Uncertainty distributions of modelled emissions (TgCO_2_eq a^−1^) for net GHG emissions of forests (NBE) assuming four harvesting scenarios (PREBAS model, areas) and anthropogenic emission scenarios WEM and WAM (FRES model, vertical bands). The simulations represent years around 2030 (top) and 2050 (bottom). PREBAS results are shown for current climate (left) and the RCP4.5 climate change scenarios (right). Positive values indicate emissions and negative values sinks. See Table 2 for definition of time periods and average and 95% uncertainty values of the scenarios. Overlapping distributions of the two models indicate decreasing probability to achieve a balance between the sources and sinks of GHGs (i.e., C neutrality) in Finland for the different scenarios. The thin vertical line for the WAM scenario in 2050 is based on assumptions of large-scale CCS/BECCS implementation (8.6 TgCO_2_eq a^−1^). See text for details

The results from the different PREBAS and FRES scenarios, considering the estimated uncertainties in the predictions, are summarised in Fig. 3. The potential to reach C neutrality (i.e., balance between the sinks and sources of GHGs), is shown as estimated uncertainty distributions of the results of the two models for two target years. The highest forest C sink values are obtained with the NoHarv scenario and increasing forest harvesting decreases the C sink values, with lowest values for the MaxHarv scenario. Overlapping distributions of the two models thus indicate decreasing probability to reach the C neutrality target for the different scenarios. Note that the assumptions of the two models regarding forest harvesting and the use of wood-based energy are for technical reasons not completely harmonised. The assumptions in the WEM and WAM scenarios are consistent with the BaseHarv scenario for which scenario the comparison of the FRES and PREBAS model results is most accurate. The NoHarv scenario is realistic only for the protected areas and is shown in Fig. 3 only for comparison. The results clearly show both the large uncertainty in the predictions and the influence of estimated climate change (RCP4.5 scenario) on the GHG balance of the forests, predicted by PREBAS (Table 2; Fig. 3). According to the PREBAS results, climate change would lead to increasing NEE and C sink values of the forests.

#### Integrated analysis of BD protection targets and C fluxes and storages of forested ecosystems

**Fig. 4:**
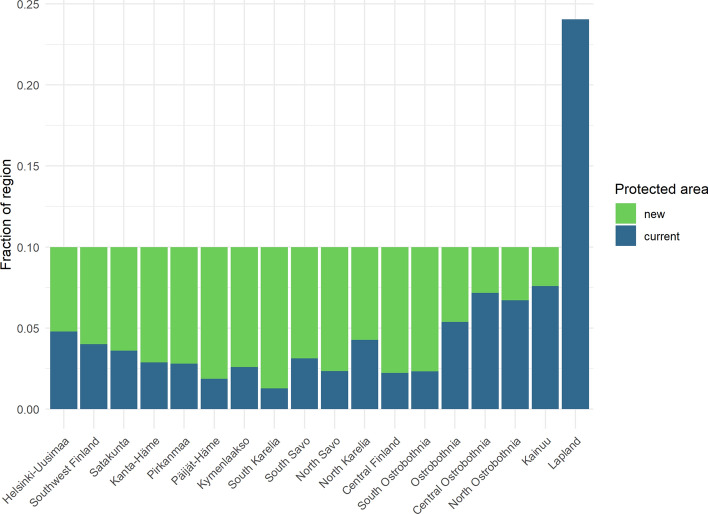
Fraction of new protected forested area needed in the 18 administrative regions of mainland Finland (see Fig. 2) to reach the 10% protection target of the EU biodiversity strategy. The target has already been reached in the Lapland region. The new protected areas were identified based on spatial prioritization of the Zonation model. See text for details

The EU BD strategy aims at an increase of strictly protected areas to 10% of the EU land area. We used the spatial prioritization tool Zonation to identify important new forested areas for BD protection in the different regions, according to the 10% strict protection target and following the approach recommended by the Finnish Nature Panel (see section on Zonation modelling above). The results indicate a large variation in the area needed to reach a regional 10% target (Fig. 4). Only in the northernmost Lapland region, this goal is already reached. A substantial increase of the protected area would be needed also in southern and eastern regions presently used for intensive forestry (e.g., South Karelia, see Fig. 2). In many of these regions, current forest harvesting is already close to sustainable wood production levels (Luke 2022).

The combined impact of applying BD protection measures and different forest harvesting scenarios on the C fluxes and storages in the forested ecosystems are shown in Table 3 (current climate conditions) and Table S3_1_1 (Supplementary Information, RCP4.5 climate scenario). In these tables the impacts of setting Zonation-prioritized forests aside as new protection area are assessed based on spatially constrained PREBAS simulations (prioritized forests excluded). The results are shown separately for the current and prioritized potential protected areas according to the 10% target, and the whole forested area. Results for the climate regulation services, defined as the ecosystem contributions to reducing concentrations of GHGs in the atmosphere (SEEA definition, see above), are also shown. The results indicate that the total average C storage (trees + ground vegetation + soil) of the currently protected forested areas is estimated to increase substantially by the mid-century, to between about 257 TgC (current climate) and 272 TgC (RCP4.5). Assuming an increase of the protected area to 10% would further increase this value (to 426–452 TgC). NBE (i.e., climate regulation service “C sequestration”) would range between -5.8 and -9.4 TgCO_2_eq a^−1^ (current area), and − 12.0 and − 17.5 TgCO_2_eq a^−1^ (10% target protection area), respectively by mid-century. The different combinations of harvesting intensity scenarios (LowHarv and MaxHarv) indicate the implications of conservation-oriented vs. intensive forestry for the managed forest land outside the areas for BD protection. It was here assumed that the harvesting intensity according to the scenarios was implemented on the reduced area until the harvest limit was reached (or as far as possible considering the age structure of the forests and other management recommendations).

**Table 3:** Area (km^2^), forest harvesting scenario, period, volume growth (Mm^3^ a^−1^), C storages in trees + ground vegetation and soils (TgC), harvested volume (Mm^3^ a^−1^), NEE (TgCO_2_eq a^−1^), climate regulation service net C sequestration (NBE) (TgCO_2_eq a^−1^) and climate regulation service C storage (TgC) of forested areas of Finland, for two levels of protected areas (currently protected areas, assuming an increase to 10% strictly protected area), and time periods. PREBAS model simulations have been made assuming current climate conditions. Results for forests are shown as averages for several years to reduce the annual variability caused by changes in climate conditions. Negative NBE values indicate sinks and positive values sources. Uncertainty estimates (95%) are shown for the NBE and C storage values. Results are shown separately for the protected areas and the whole forested area (protected + managed area) of Finland. For the protected areas (currently protected and 10% target areas), the NoHarv scenario is assumed. For the whole area, the results of three harvesting intensity scenarios (BaseHarv, LowHarv and MaxHarv) for the forested areas outside the currently strictly protected areas are shown. These harvesting intensity scenarios indicate the implications of intensive vs. conservation-oriented forestry for the managed forest land with respect to key C variables. The climate regulation services net C sequestration (NBE) and C storage were calculated according to SEEA definitions. Corresponding results assuming climate change scenario RCP4.5 are shown in Table S3_1 (Supplementary Information). See text for details

	Area (km^2^)	Scenario	Period	Volume growth (Mm^3^ a^−1^)	C storage above ground (TgC)	C storage soils (TgC)	Harv. volume (Mm^3^ a^−1^)	NEE (TgCO_2_eq a^−1^)	Climate serv. net C sequestration (NBE) (95%) (TgCO_2_eq a^−1^)	Climate serv. C storage (95%) (TgC)
Currently protected	16 428	NoHarv	2017–2025	4.8	54.1	176.8	0	− 6.1	− 6.1 (− 6.9, − 5.2)	230.9 (217.1, 243.9)
Currently protected	16 428	NoHarv	2034–2050	4.7	79.3	177.4	0	− 5.8	− 5.8 (− 6.7, − 4.8)	256.7 (239.6, 272.3)
10% Target	27 053	NoHarv	2017–2025	12	130.6	245.6	0	− 14.4	− 14.4 (− 15.9, − 12.4)	376.2 (352.5, 394.2)
10% Target	27 053	NoHarv	2034–2050	10.6	180.4	245.8	0	− 12	− 12 (− 13.4, − 10.3)	426.2 (406.8, 442.4)
Whole region	211 130	BaseHarv	2017–2025	107.5	924.4	2617	81.5	− 87.8	− 28 (− 43.7, − 10.4)	3541.4 (3350.5, 3704.4)
Whole region	211 130	BaseHarv	2034–2050	77.1	850.1	2547	81.3	− 45.2	15.9 (− 0.1, 34.2)	3397.1 (3176.6, 3556.1)
Whole region	211 130	LowHarv	2017–2025	107.9	934.3	2616.7	67	− 89.7	− 40.8 (− 55.8, − 23.1)	3551 (3360.6, 3714)
Whole region	211 130	LowHarv	2034–2050	90.3	1070.7	2575.3	50.9	− 67.7	− 31.4 (− 43.9, − 15.7)	3646 (3477.4, 3784.6)
Whole region	211 130	MaxHarv	2017–2025	107.2	912.3	2613	88.7	− 85.1	− 20.1 (− 35.9, − 2)	3525.3 (3336, 3686.6)
Whole region	211 130	MaxHarv	2034–2050	71.3	721.1	2525.5	89.9	− 35.1	32.9 (19.2, 47.1)	3246.6 (3044.4, 3399.9)

**Fig. 5:**
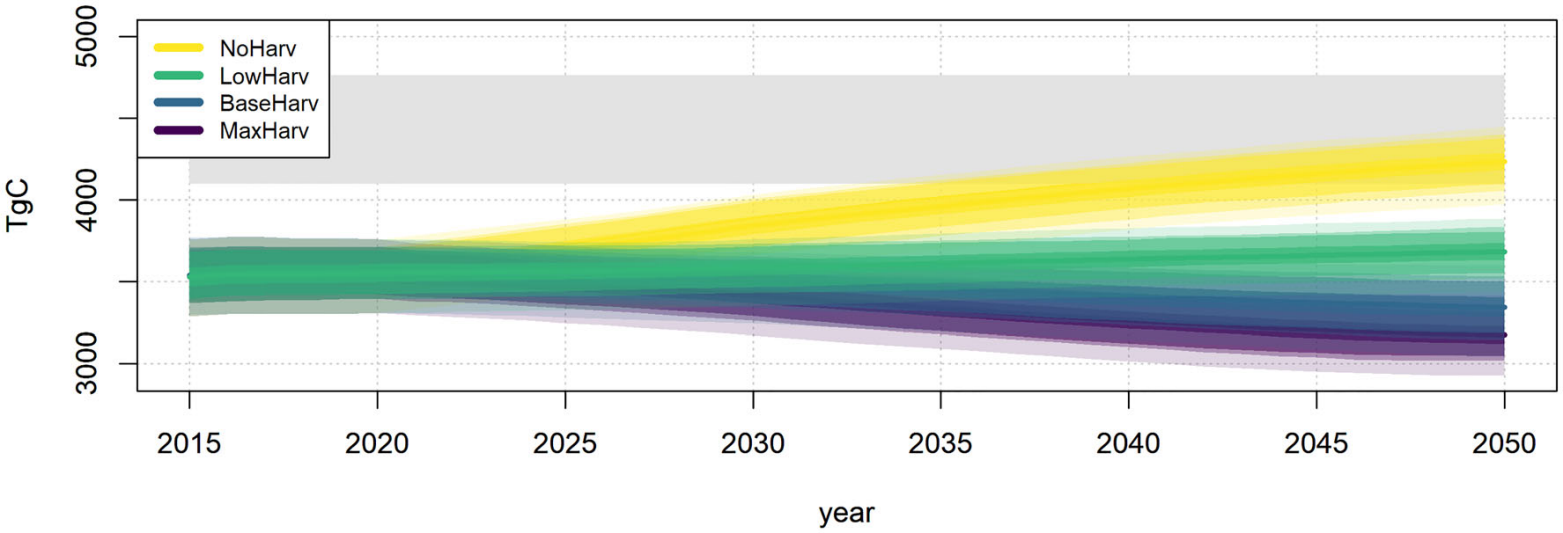
Time development of the estimated carbon storage in the forested ecosystem (trees + ground vegetation + soil, TgC) in Finland assuming different forest harvesting intensity scenarios (PREBAS model, see Table 2), compared with the estimated maximum C storage potential (horizontal shaded grey area). The model runs have been made assuming current climate conditions and uncertainty estimates of the scenarios are shown. The maximum C storage potential was estimated by running the PREBAS model with the NoHarv scenario for 100 years until 2120

We also used PREBAS to estimate the theoretical maximum C storage potential of Finnish forest ecosystems, by running the model assuming a long-term NoHarv scenario (Fig. 5). The results clearly indicate that particularly the more intensive harvesting scenarios result in a long-term C storage far below this maximum of some 4500 TgC (Fig. 5). Note that the NoHarv scenario is here assumed for the whole forested area (not only for the protected areas). The intensive MaxHarv scenario would result in a decreasing C storage by mid-century also assuming the RCP4.5 scenario (Table S3_1). This approach thus indicates the long-term potential of the climate regulation services. Assuming a C price of about 70 € t^−1^ for anthropogenic CO_2_ emissions according to the current level of the EU ETS emission trading system (and counting for the change from CO_2_ to C), the total economic value of this potential maximum C storage would be about 1 150 000 M€. For the estimated 10% protection area, the corresponding values by mid-century would range between ca. 109 000 M€ (current climate scenario) and 116 000 M€ (RCP4.5 scenario, Table 3; Table S3_1). These results thus provide background information for optimizing use of climate regulation services in national and regional climate change mitigation planning. The economic value of the forest industry products in Finland was about 18 000 M€ in year 2021 (Ministry of Agriculture and Forestry).

### Regional estimates

**Fig. 6:**
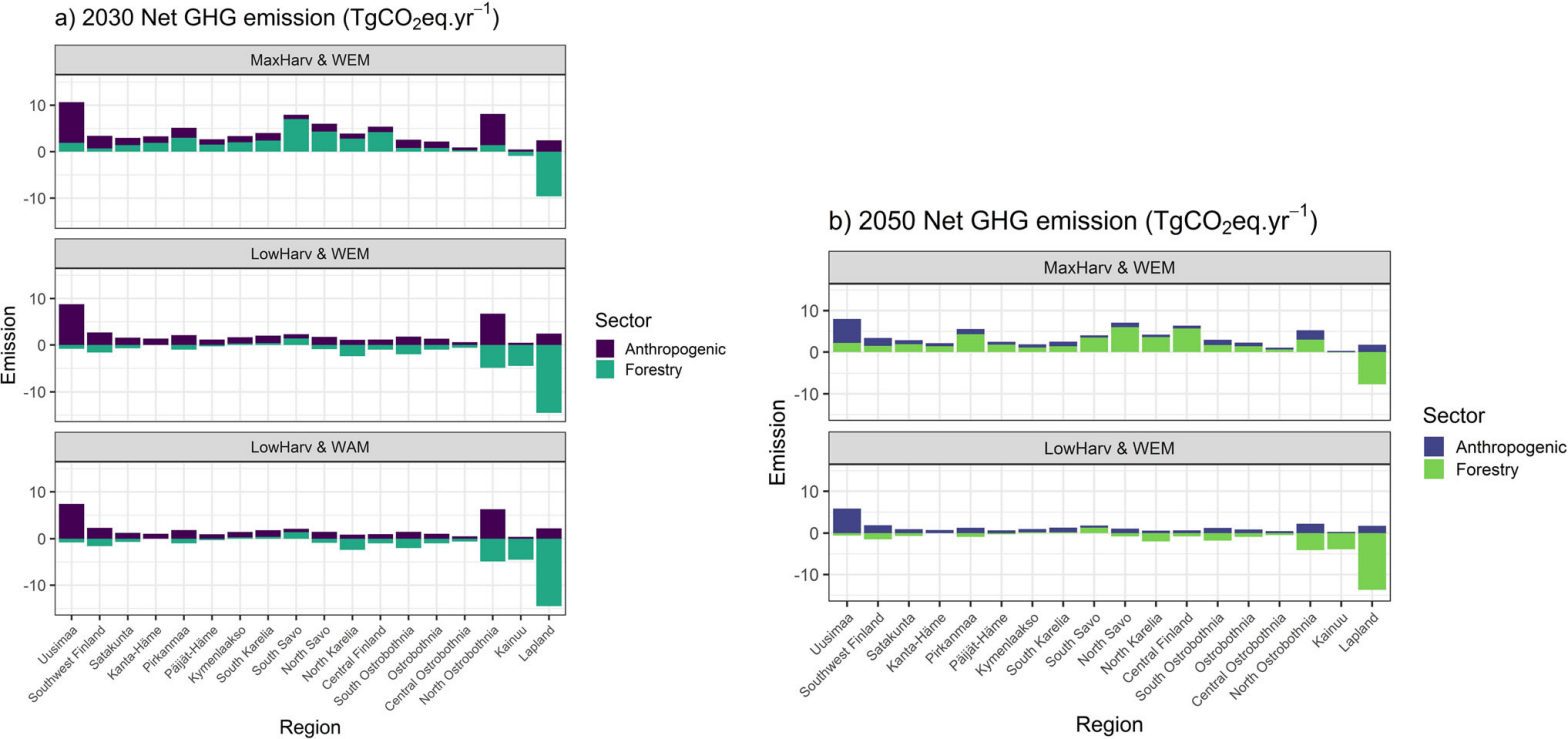
Net GHG emissions in 2030 (**a**) and 2050 (**b**) in each of the 18 regions of mainland Finland (see Fig. 2). Two forest harvesting scenarios (LowHarv, MaxHarv) were combined with two anthropogenic scenarios (WEM, WAM) for emissions in 2030 (**a**). For 2050 net emissions (**b**), only one anthropogenic scenario (WEM) is shown with both the forestry scenarios (LowHarv, MaxHarv) because the partitioning of the WAM emissions into the regions was not relevant for 2050. Positive values indicate emissions and negative values sinks (TgCO_2_eq a^−1^). Current climate conditions assumed. The results for forests are presented as the average for several years to account for the impacts of variations in weather conditions. See text for details

The model results were aggregated also separately for the 18 administrative regions of mainland Finland (Fig. 2), to provide scientific data support also for regional decision-making. The detailed results are presented in Tables S2_2–S2_3, S3_2–S3_3 and S4 (Supplementary Information). Figure 6 summarizes the results regarding the net GHG emissions of three different combinations of the scenarios, including the high and low end of these combinations. The results refer to results around the years 2030 and 2050 for the anthropogenic emissions (FRES model) and forests (PREBAS model, current climate conditions). The results clearly show the significant differences between the estimated net GHG budgets of the regions, and in the potential for reaching regional C neutrality. Moreover, the need for regional cooperation in supporting national policy goals is clearly indicated. The largest net GHG sinks were in the northern regions (North Ostrobothnia, Kainuu and Lapland), where the average values for the LowHarv scenario ranged between − 4.9 and − 14.5 TgCO_2_eq a^−1^ around 2030 (current climate scenario). The MaxHarv scenario would cause the forestry sector to be an emission source in most regions (Fig. 6). The range in anthropogenic emissions of the 18 regions was also large, between 0.4 and 7.4 TgCO_2_eq a^−1^ for the WAM scenario in 2030 (Table S4).

## Discussion

### Current GHG balances and C storages of different land-use classes and ecosystem compartments

Quantitative data on the distribution of GHG fluxes and C storages of the different land use classes and ecosystem compartments is important for understanding the element cycles, analyzing impacts of land-use and conservation activities, and for the design of climate change mitigation measures (IPCC 2022; Holmberg et al. 2023). As shown in Table 1, anthropogenic emissions from artificial surfaces are currently clearly the largest source of GHG emissions and the forested areas are the largest sink in Finland. The C storages are dominated by the peatland areas, forest soils and tree and ground vegetation biomass. Emissions from peatlands drained for forestry and used for agriculture are important GHG emission sources and have received attention in national GHG mitigation plans (Ojanen et al. 2010; Finnish Climate Change Panel 2021). Cultivated organic soils are a major source of agricultural GHG emissions although they cover only 10% of the field area in Finland (Regina et al. 2019). Similarly, the total CO_2_, N_2_O and CH_4_ emission from forestry drained peatland in 2020 were 7.5 TgCO_2_eq a^−1^. Thus, these emissions are important in terms of the national GHG budget (Statistics Finland 2021). Different land use activities of Finnish peatlands have reduced the total peat C storage by 3–10% (172–510 Tg) since 1950 (Turunen and Valpola 2020).

The surface area of lakes in in Finland is about 10% of the land area, and lakes and rivers are an important GHG emission source due to degradation of organic matter derived mainly from terrestrial sources (13.4 TgCO_2_eq a^−1^, Table 1). Lake sediments are also a large C storage, which has accumulated since the last glaciation (Table 1, Kortelainen et al. 2004). Leaching of dissolved organic and inorganic carbon (DOC and DIC) from the terrestrial area into the river network is thus a key component of the areal C budget (Kortelainen et al. 2013; Gommet et al. 2022). Finnish rivers export annually on average about 1.2 TgC to the Baltic Sea (Räike et al. 2016). However, surface waters are currently not explicitly considered in the Finnish GHG accounting system.

### Impacts of anthropogenic emission, forest harvesting and climate change scenarios on net GHG budgets

Our GHG scenario analyses were focused on the anthropogenic sources and forested areas as they are the two most significant GHG sources and sinks in the landscape (Table 1) and are directly influenced by mitigation and management measures. As shown in Table 2, the estimated average anthropogenic GHG emissions in Finland by year 2050 (22.9 and 8.6/17.6 TgCO_2_eq a^−1^ for WEM and WAM scenarios, respectively) indicate a significant reduction from the current level of anthropogenic GHG emissions, 47.7 TgCO_2_eq a^−1^ in 2021 (Statistics Finland 2022). The minimum value of the WAM scenario in 2050 (8.6 TgCO_2_eq a^−1^) assumes large-scale implementation of CCS/BECCS measures after 2040, which have not been tested at this magnitude before. This obviously introduces an additional element of uncertainty in this scenario. Moreover, the impacts of the different forest harvesting intensity scenarios and climate change scenarios on the net GHG emissions (NBE), predicted by PREBAS, are also shown (Table 2). According to the Finnish Climate Change Panel the C sink of the forests should be about − 27 TgCO_2_eq a^−1^ by year 2035 to reach the governmental C neutrality target (Finnish Climate Change Panel 2019), and the EU regulations require that the Finnish net GHG sink of the whole LULUCF sector should be at least − 17.8 TgCO_2_eq a^−1^ by 2030 (EU 2021b).

According to our PREBAS scenario results, the probability of reaching the overall policy target of C neutrality by 2035 (and net negative GHG emissions thereafter) is influenced by the assumptions on the impacts of climate change (Fig. 3). If a continuation of current climate is assumed, reaching this target in both 2035 and 2050 would be challenging, and a combination of the WAM scenario for the anthropogenic GHG emissions and forest harvesting closer to the LowHarv scenario would be needed (Fig. 3; Table 2). Realized total harvesting levels are higher than in the LowHarv scenario, about 79 Mm^3^ a^−1^ in 2021. It should also be noted that we are at present not modelling future developments of GHG emissions from arable land, which currently form a significant emission source (12.2 TgCO_2_eq a^−1^, Table 1) in the LULUCF sector. These emissions have been stable during recent years, indicating that a net sink from the forests would be needed to compensate also for these emissions.

PREBAS modelling with the climate change scenario RCP4.5 results in a higher net GHG sink of the forests, implying more flexibility in reaching the C neutrality goal (Fig. 3). PREBAS modelling assuming the high-change RCP8.5 scenario indicates even larger increases in NEE (Junttila et al. 2023). There is, however, considerable uncertainty in these estimates, because several factors potentially reducing future forest growth and volumes are not considered in PREBAS (see below). The estimated decreasing future C sequestration capacity of the forests in the current climate situation is mainly due to the present age structure of the forests, combined with the forest management (see Junttila et al. 2023; Mäkelä et al. 2023). Forest growth has been increasing for a long period while harvests have been lower than growth, leading to ageing of stands on average and higher average per hectare growing stocks. This effect is seen also in the LULUCF calculations of the most recent national GHG inventory (Statistics Finland 2022). Reaching C neutrality needs to be assisted by actions also in other LULUCF sectors, and by general energy efficiency and production measures (Finnish Climate Change Panel 2019; Saikku et al. 2022).

### Integrated GHG and BD scenario analyses

We used the Zonation software for identification of the forested areas best suitable for increasing the protected area to 10% in the different regions, according to the EU and Finnish Nature Panel targets (Fig. 4) and estimated the C sequestration and storages using PREBAS modelling. Our results are consistent with those from international studies. Although stem growth declines with the ageing of trees, old-growth forests store substantial amounts of C and can continue to sequester C in aboveground biomass and soils for hundreds of years (Luyssaert et al. 2008; Gundersen et al. 2021; Stokland 2021). The total C storage (trees + ground vegetation + soil) of the currently protected forested areas was estimated to increase substantially by the mid-century (257 TgC for current climate and 272 TgC assuming RCP4.5). Increasing the protected area according to the 10% target would further increase this value (426–452 TgC). These areas would also provide a significant C sink, NBE ranging between − 5.8 and − 9.4 TgCO_2_eq a^−1^ (current area), and − 12.0 and − 17.5 TgCO_2_eq a^−1^ (10% area), respectively by mid-century (Table 3; Table S3_1). As shown in Fig. 5, a continued long-term increase of the C storage of the whole forested area would occur also in the future, assuming low forest harvesting.

Table 3 and Table S3_1 show the results where two harvesting scenarios (LowHarv and MaxHarv) for the forested areas outside the strictly protected areas are assumed. These harvesting scenarios indicate the implications of conservation-oriented vs. intensive forestry for the managed forest land with respect to key C related variables. According to Blattert et al. (2022), the separation of the forest landscape into clearly protected and more extensively managed areas for production purposes, follows a land sparing or segregation approach. By allocating the land to areas with different management purposes, conflicts will be minimized and the overall multifunctionality of the forest landscape will increase. The integrated impact of the protection measures and harvesting scenarios on the total C storages and net GHG emissions of the forested areas is thus dependent on both the protection measures and the forest harvesting intensity outside the protected areas (see Mäkelä et al. 2023).

### Assessing climate regulation services of forested ecosystems

There has been a strong quest for mapping and assessing ES to support governance (Primmer et al. 2021). Efforts to link this information to tracking changes in ecosystem assets and accounting for environmental externalities are increasing, aiming at developing better systems for sustainable economics (Dasgupta 2021). As outlined above, “climate regulation services” have been defined to estimate ecosystem contributions to reducing concentrations of GHGs in the atmosphere (EU 2021a; UN 2021). We estimated the service values “net C sequestration” and “C storage” for the forested ecosystems as part of our PREBAS modelling exercise, to demonstrate the concept and its connection to the BD protection measures and forest harvesting scenarios (Table 3; Table S3_1; Fig. 5).

As demonstrated in Fig. 5, there is a large potential to store C in Finnish forest ecosystems, the theoretical maximum amounting to about 4500 TgC. The potential economic value of this maximum C storage is significant, also for the protected areas. Particularly the more intensive harvesting scenarios indicate C storage far below this maximum potential. It should, however, be recognized that different environmental factors, such as forest fires, nutrient limitations and insect attacks influence the possibilities to maintain this maximum potential for extended time periods (Anderegg et al. 2020; Akselsson et al 2021; Belyazid et al. 2022). In addition, the forest industry is of substantial importance for the national economy, and therefore extensive forest management is foreseen also in the future. Still, our results clearly demonstrate the potential for integrated C and BD management in these forested systems. In addition to protection, measures supporting BD in managed forests, such as increasing the amount of deadwood and continuous cover forestry, provide additional benefits (e.g., Tikkanen et al. 2012; Forsius et al. 2016; Blattert et al. 2022; Kuusela et al. 2022; Mäkelä et al. 2023). Such integrated plans can be supported by implementation of economic instruments, such as a PES (payment for ESs) scheme promoting both forest BD conservation and C sequestration/storage (Kangas and Ollikainen 2022). This is particularly important in more southern Finnish regions, where a substantial proportion of the forest land is privately owned. Connecting a C premium to the current METSO-program for BD protection of Finnish forest ecosystems could support these multiple goals in a socially efficient manner (Kangas and Ollikainen 2022).

ES accounting will be mandatory for the EU countries, and C storage and sequestration are key ESs to be considered. Limiting global-mean temperature rise to 1.5–2 °C according to the international targets requires large net negative GHG-emissions on the global scale (Rogelj et al. 2019; IPCC 2022), and also in Finland long-term mitigation targets require net negative GHG emissions (Finnish Climate Change Panel 2021). Halting the loss of BD and ESs is on both the global (Diaz et al. 2019) and Finnish national agendas. These are gigantic challenges, putting large demands on future land use policies, energy production systems and technological solutions for GHG-capturing from the atmosphere. We show how integrated modelling of BD conservation measures, GHG budgets and ESs can provide information for actions on both national and regional scales.

### Aggregating results to administrative regions

Our results for the 18 administrative regions (Figs. 4, 6; Tables in Supplementary Information) aim at supporting implementation of regional climate roadmaps and integrated BD protection measures. The regions will receive our scenario results also as gridded spatial information, to support detailed land- and energy-use planning. Many regions have ambitious plans to implement such measures, and the role of regional actors such as regional councils, ELY Centers, and development organizations in achieving these objectives is essential (Saikku et al. 2022). The significant differences between the regions in their potential to achieve regional-scale C neutrality and a regional 10% BD target are clearly indicated (Figs. 4, 6), also emphasizing the need for regional cooperation in supporting national-scale targets. Our results indicate that intensive forest harvesting clearly would make it exceedingly difficult to reach C neutrality in many regions on both shorter and longer time scales (Fig. 6). There are currently plans to establish new forest industry plants in the northern regions, where the largest harvesting potential currently is unused (Fig. 6). This would have consequences for the implementation of the national climate change mitigation plans. The need for implementing different policy instruments to support both regional and national C neutrality and BD goals is also evident (Kangas and Ollikainen 2022). The impacts of climate change on the regional-scale PREBAS results are consistent with those for the whole country.

### Limitations

We recognize the large uncertainties involved in our regional-scale modelling approach. These can be roughly grouped into two categories: (i) uncertainties owing to model structure (conceptualization), and (ii) uncertainties in the model parameters and input data. We have tried to deal with the latter issue by introducing uncertainty into the model parameter values (see “Materials and methods” section). A thorough evaluation of the uncertainties concerning PREBAS applications and C budget calculations is available in Minunno et al. (2019) and Junttila et al. (2023). Previous model comparisons also reveal that currently used forest models for many reasons do not always give consistent results (van Oijen et al. 2013). Uncertainties in Zonation based modelling are dealt with in detail in Kujala et al. (2023), but, e.g., the point-nature of species data is of concern in landscape level analyses. The choice of data resolution is a major issue and our approach within the current study was pragmatic; we used the best available data (e.g., the MS-NFI, national GHG emission databases and species inventories). The PREBAS model has also been developed and calibrated using highest-class data from intensively studied research sites (Minunno et al. 2016, 2019). Another prominent issue is that the current PREBAS model version does not consider nutrient limitations (N, P, base cations) and changes in e.g., forest fires, drought frequencies and insect attacks, potentially reducing forest growth and biomass in a climate change situation (Venäläinen et al. 2020; Belyazid et al. 2022). The PRELES submodule of PREBAS includes a simple bucket model of soil water balance, which influences evapotranspiration and photosynthesis through a simplified description of stomatal control, but the issue of water availability would still need further work. Therefore, the PREBAS results in a climate change situation are likely on the high end of the distribution, particularly on less fertile soils (see Norby et al. 2010). The forest harvesting levels are also influenced by regional demand and thus not necessarily always realistic.

There are numerous factors that bring uncertainty to the calculation and spatial allocation of anthropogenic GHG emissions. Many of these factors, especially those related to the location of emissions, are difficult to quantify. In the FRES model, point source emissions are calculated representing average fuel mixes and operating hours for combustion plants and average emissions for process industry plants over the years 2018–2020. However, for many of the plants, annual variation is large, and some of the plants may not even be in use in a given year. Therefore, although the country total emissions of a certain point source sector can be quantified (Holmberg et al. 2023), the uncertainty of emissions for individual plants (and thus for the location of emissions) is higher. Spatial distribution uncertainties were not quantitatively assessed. The uncertainty for small scale wood combustion has been addressed in Paunu et al. (2021), where it was concluded that the country level data and methods can produce similar spatial allocation as a local level bottom-up inventory, increasing the confidence in the method used in our assessment.

For the scenario years 2030 and 2050, the locations of point sources in 2019 were used; therefore, future changes in emissions spatial distribution due to e.g., relocation of industrial plants or population (urbanization) are not covered. The reduction in emissions is due to an overall reduction in fossil fuel use. In the HIISI scenario WAM 2050, CCS and BECCS are assumed to play a major role in reduction of GHG emissions. As there is no information on the regional application of these technologies, we have not included them in the spatial allocation of emissions. In a more detailed regional approach, a bottom-up method with plans for each facility would be needed. Due to the ambiguity of received HIISI data to the FRES model, the GHG emission calculation could not be exactly reproduced in the two models. Total CO_2_eq emissions calculated with the FRES model were slightly lower than HIISI emissions for the WEM scenario (1% in 2030 and 3% in 2050) and slightly higher than HIISI emissions for the WAM scenario (8% in 2030 and 6% in 2050).

## Conclusions

Reaching C neutrality and mitigating climate change at global, national, and regional scales is an enormous challenge for human society. Achieving this target requires quantitative data on the distribution and future developments of GHG fluxes and C storages of key sectors and ecosystem compartments. Our scenario results for Finland indicated that the probabilities to reach the overall policy target of C neutrality by 2035 (and net negative GHG emissions thereafter) is influenced by the assumptions on the impacts of climate change on future forest growth. Assuming continuation of the current climate, the C/GHG target could only be reached by a combination of strong mitigation measures of the anthropogenic GHG emissions and low forest harvesting intensity (clearly below the current averages). Strong emission mitigation measures and modelled increasing forest growth due to climate change would potentially make these climate targets less demanding, particularly by the mid-century. The large uncertainty in these estimates should be recognized, and uncertainties should also be more thoroughly considered in national decision-making. The estimated future net GHG balances in the different administrative regions of Finland varied greatly, due to differences in the distribution of anthropogenic point and areal emission sources, forest resources and forest harvesting intensities. Our results thus emphasize the need for regional cooperation in reaching national climate targets. We also show that potential new forested areas for BD protection according to the EU and national 10% protection target would provide a significant present and estimated future C storage and C sequestration potential. The estimated GHG sequestration potential of the protected forested areas according to the 10% target by mid-century (about − 12 to − 17.5 TgCO_2_eq a^−1^ assuming current climate and RCP4.5 conditions, respectively) could be an almost sufficient GHG net sink to compensate for both the remaining anthropogenic emissions according to the (optimistic) WAM scenario in 2050 (8.6 TgCO_2_eq a^−1^) and GHG emissions from agricultural soils (currently 12.2 TgCO_2_eq a^−1^, Table 1). This indicates complementarity of mitigation and conservation measures and indicates the need to better integrate climate and BD policies. However, we also show that the overall future net GHG emissions of the forested areas (protected and managed) obviously are determined by both the assumed protection measures and the harvesting intensity in the managed areas (Table 3). The C sinks and storages of these areas are also important ESs for climate regulation and have potentially large economic value. This ES provision can be supported by implementation of integrated PES (payments for ES) schemes. Accounting of ESs is a rapidly growing field and will soon be a mandatory process in the EU countries. Our results demonstrate the potential and provide information for integrated GHG, BD and ES management and evaluation of forested ecosystems and regions.

### Supplementary Information

Below is the link to the electronic supplementary material.

Supplementary file1 (PDF 1143 kb)
