# Supplementary material for: Modelling the regional potential for reaching carbon neutrality in Finland: Sustainable forestry, energy use and biodiversity protection
Source: Ambio. 2023 Aug 10;52(11):1757–76. doi: 10.1007/s13280-023-01860-1 (PMC10562359; doi:10.1007/s13280-023-01860-1)
Supplement: Supplementary file 1 — Supplementary file1 (PDF 1143 kb) [file 13280_2023_1860_MOESM1_ESM.pdf]

Ambio

Electronic Supplementary Material

Title: **Modelling the regional potential for reaching carbon neutrality in Finland: sustainable forestry, energy use and biodiversity protection**

Authors: Martin Forsius, Maria Holmberg, Virpi Junttila, Heini Kujala, Torsti Schulz, Ville-Veikko Paunu, Mikko Savolahti, Francesco Minunno, Anu Akujärvi, Jaana Bäck, Juha Grönroos, Risto K. Heikkinen, Niko Karvosenoja, Annikki Mäkelä, Ninni Mikkonen, Minna Pekkonen, Katri Rankinen, Raimo Virkkala

The following tables provide additional information:
S2_2 (18 regions, current climate scenario)
S2_3 (18 regions, RCP4.5 climate scenario)
S3_1 (national scale, RCP4.5 climate scenario)
S3_2 (18 regions, current climate scenario)
S3_3 (18 regions, RCP4.5 climate scenario)
S_4 (FRES model scenarios for anthropogenic emissions for the 18 regions)

Table S2_2. Impact of forest harvesting intensity and climate change scenarios (PREBAS model, current climate conditions) on aggregated GHG emissions and sinks ($TgCO_2eq\ a^{-1}$) for forested areas of the 18 administrative regions of mainland Finland (see Fig. 2 in main paper). The forest harvesting intensity scenarios (BaseHarv, LowHarv, MaxHarv) are based on the national climate and energy strategy and forestry policies. For forests, emissions are assumed to equal harvested biomass and sinks NEE (net ecosystem exchange). The net emissions of forested areas (NBE) were calculated by adding NEE estimates to the amount of harvested biomass and considering CH_4 and N_2O flux from organic soils. Positive values indicate emissions and negative values sinks. Results for forests are shown as averages for several years to reduce the annual variability caused by changes in climate conditions. The present situation refers to average result for years 2017-2025. The best available estimates of uncertainty (95%) are also shown for NEE and NBE. Corresponding results assuming climate change scenarios RCP4.5 are shown in Table S2_3. See main paper for details.

Region	Scenario	Period	Harvested volume $Mm^3\ a^{-1}$	Harvested biomass $TgCO_2eq\ a^{-1}$	NEE (95%) $TgCO_2eq\ a^{-1}$	NBE (95%) $TgCO_2eq\ a^{-1}$
01 Uusimaa	BaseHarv	2017 - 2025	4	3	-3.6 (-4.1, -2.8)	-0.6 (-1.1, 0)
	BaseHarv	2026 - 2033	4.3	3.3	-2.4 (-3.3, -1.5)	0.8 (0, 1.8)
	BaseHarv	2034 - 2050	4.2	3.2	-1.5 (-2.5, -0.5)	1.7 (0.8, 2.6)
	LowHarv	2017 - 2025	3.3	2.4	-3.6 (-4, -2.9)	-1.2 (-1.7, -0.6)
	LowHarv	2026 - 2033	2.6	1.9	-2.7 (-3.2, -2.2)	-0.8 (-1.3, -0.4)
	LowHarv	2034 - 2050	2.5	1.8	-2.5 (-2.9, -1.9)	-0.7 (-1.1, -0.1)
	MaxHarv	2017 - 2025	4.4	3.2	-3.5 (-4, -2.7)	-0.3 (-0.8, 0.3)
	MaxHarv	2026 - 2033	5.1	3.9	-2.1 (-3, -1.1)	1.9 (1, 2.9)
	MaxHarv	2034 - 2050	4.2	3.3	-1.1 (-1.9, -0.4)	2.2 (1.5, 2.7)
02 Southwest Finland	BaseHarv	2017 - 2025	3.6	2.6	-4 (-4.6, -3.2)	-1.4 (-2.1, -0.7)
	BaseHarv	2026 - 2033	3.7	2.8	-2.9 (-3.7, -2.1)	-0.2 (-0.9, 0.6)
	BaseHarv	2034 - 2050	3.7	2.8	-2.3 (-3, -1.3)	0.5 (-0.1, 1.5)
	LowHarv	2017 - 2025	2.9	2.1	-4.1 (-4.6, -3.3)	-1.9 (-2.5, -1.2)
	LowHarv	2026 - 2033	2.2	1.6	-3.3 (-3.8, -2.8)	-1.7 (-2.2, -1.1)
	LowHarv	2034 - 2050	2.2	1.6	-3.1 (-3.6, -2.5)	-1.5 (-2, -1)
	MaxHarv	2017 - 2025	3.9	2.8	-4 (-4.6, -3.2)	-1.1 (-1.7, -0.4)
	MaxHarv	2026 - 2033	4.5	3.3	-2.6 (-3.4, -1.8)	0.7 (0, 1.6)
	MaxHarv	2034 - 2050	4.4	3.4	-1.8 (-2.6, -0.8)	1.5 (0.8, 2.3)
04 Satakunta	BaseHarv	2017 - 2025	3.1	2.3	-2.8 (-3.3, -2.3)	-0.6 (-1, 0)
	BaseHarv	2026 - 2033	3.2	2.4	-1.8 (-2.4, -1.2)	0.6 (0, 1.2)
	BaseHarv	2034 - 2050	3.2	2.4	-1.3 (-1.9, -0.6)	1.1 (0.6, 1.8)
	LowHarv	2017 - 2025	2.5	1.8	-2.9 (-3.3, -2.3)	-1 (-1.4, -0.5)
	LowHarv	2026 - 2033	1.9	1.4	-2.1 (-2.6, -1.7)	-0.8 (-1.2, -0.3)
	LowHarv	2034 - 2050	1.9	1.3	-2.1 (-2.5, -1.6)	-0.7 (-1.1, -0.2)
	MaxHarv	2017 - 2025	3.4	2.5	-2.7 (-3.2, -2.1)	-0.3 (-0.7, 0.2)
	MaxHarv	2026 - 2033	3.8	2.9	-1.5 (-2.1, -0.9)	1.4 (0.8, 1.9)
	MaxHarv	2034 - 2050	3.7	2.8	-0.9 (-1.5, -0.1)	1.9 (1.4, 2.5)
05	BaseHarv	2017 - 2025	2.7	2	-1.7 (-2, -1.3)	0.3 (0, 0.6)

Kanta-Häme	BaseHarv	2026 - 2033	2.8	2.1	-1 (-1.4, -0.5)	1.2 (0.8, 1.6)
	BaseHarv	2034 - 2050	2.6	2	-0.5 (-1, -0.1)	1.5 (1.1, 1.8)
	LowHarv	2017 - 2025	2.2	1.6	-1.7 (-2, -1.3)	-0.1 (-0.4, 0.3)
	LowHarv	2026 - 2033	1.7	1.2	-1.2 (-1.5, -0.9)	0 (-0.3, 0.3)
	LowHarv	2034 - 2050	1.7	1.2	-1.2 (-1.5, -0.8)	0 (-0.3, 0.4)
	MaxHarv	2017 - 2025	2.9	2.1	-1.6 (-1.9, -1.2)	0.5 (0.2, 0.8)
	MaxHarv	2026 - 2033	3.4	2.6	-0.7 (-1.1, -0.3)	1.9 (1.5, 2.3)
	MaxHarv	2034 - 2050	2.3	1.8	-0.4 (-0.7, -0.1)	1.4 (0.7, 1.8)
06<br>Pirkanmaa	BaseHarv	2017 - 2025	6.3	4.6	-5.3 (-6.2, -4.2)	-0.7 (-1.5, 0.3)
	BaseHarv	2026 - 2033	6.5	4.9	-3.5 (-4.6, -2.3)	1.4 (0.3, 2.5)
	BaseHarv	2034 - 2050	6.5	5	-2.4 (-3.6, -0.7)	2.6 (1.3, 4.3)
	LowHarv	2017 - 2025	5.2	3.7	-5.3 (-6.1, -4.2)	-1.6 (-2.4, -0.5)
	LowHarv	2026 - 2033	3.9	2.8	-3.9 (-4.8, -3)	-1.1 (-1.9, -0.2)
	LowHarv	2034 - 2050	3.9	2.8	-3.7 (-4.4, -2.8)	-0.9 (-1.7, 0)
	MaxHarv	2017 - 2025	6.8	5	-5.2 (-6, -4.1)	-0.2 (-1, 0.9)
	MaxHarv	2026 - 2033	7.8	5.9	-2.9 (-4.1, -1.7)	3 (1.9, 4.2)
	MaxHarv	2034 - 2050	7.5	5.8	-1.5 (-2.9, 0)	4.3 (3.2, 5.2)
07<br>Päijät-Häme	BaseHarv	2017 - 2025	2.9	2.1	-2.2 (-2.5, -1.7)	-0.1 (-0.5, 0.4)
	BaseHarv	2026 - 2033	3	2.3	-1.4 (-1.9, -1)	0.8 (0.4, 1.3)
	BaseHarv	2034 - 2050	2.9	2.3	-1 (-1.5, -0.3)	1.3 (0.8, 1.9)
	LowHarv	2017 - 2025	2.4	1.7	-2.2 (-2.6, -1.7)	-0.5 (-0.8, 0)
	LowHarv	2026 - 2033	1.8	1.3	-1.6 (-2, -1.3)	-0.3 (-0.7, 0.1)
	LowHarv	2034 - 2050	1.8	1.2	-1.6 (-1.9, -1.2)	-0.3 (-0.6, 0.1)
	MaxHarv	2017 - 2025	3.1	2.3	-2.1 (-2.5, -1.6)	0.1 (-0.2, 0.6)
	MaxHarv	2026 - 2033	3.6	2.7	-1.2 (-1.6, -0.7)	1.5 (1.1, 2)
	MaxHarv	2034 - 2050	3.1	2.4	-0.6 (-1.1, -0.2)	1.8 (1.3, 2.1)
08<br>Kymenlaakso	BaseHarv	2017 - 2025	2.3	1.7	-1.4 (-1.6, -1)	0.4 (0.1, 0.7)
	BaseHarv	2026 - 2033	2.5	1.9	-0.6 (-1.1, -0.2)	1.3 (0.8, 1.8)
	BaseHarv	2034 - 2050	2.1	1.6	-0.3 (-0.6, 0)	1.3 (0.8, 1.6)
	LowHarv	2017 - 2025	1.9	1.4	-1.4 (-1.7, -1)	0 (-0.3, 0.3)
	LowHarv	2026 - 2033	1.5	1.1	-0.8 (-1.2, -0.5)	0.3 (-0.1, 0.6)
	LowHarv	2034 - 2050	1.5	1.1	-0.9 (-1.2, -0.6)	0.2 (-0.1, 0.6)
	MaxHarv	2017 - 2025	2.5	1.9	-1.3 (-1.6, -0.9)	0.6 (0.3, 0.9)
	MaxHarv	2026 - 2033	3	2.3	-0.3 (-0.8, 0.1)	2 (1.5, 2.4)
	MaxHarv	2034 - 2050	1.7	1.3	-0.3 (-0.5, 0)	1.1 (0.4, 1.5)
09<br>South Karelia	BaseHarv	2017 - 2025	2.9	2.2	-1.7 (-2, -1.2)	0.5 (0.2, 0.9)
	BaseHarv	2026 - 2033	3.1	2.4	-0.8 (-1.4, -0.2)	1.6 (1.1, 2.2)
	BaseHarv	2034 - 2050	2.6	2	-0.3 (-0.8, 0.1)	1.7 (1.1, 2)
	LowHarv	2017 - 2025	2.4	1.8	-1.7 (-2.1, -1.3)	0.1 (-0.3, 0.4)
	LowHarv	2026 - 2033	1.9	1.4	-1 (-1.5, -0.6)	0.3 (-0.1, 0.8)
	LowHarv	2034 - 2050	1.8	1.3	-1.1 (-1.4, -0.7)	0.2 (-0.1, 0.7)
	MaxHarv	2017 - 2025	3.1	2.4	-1.6 (-1.9, -1.1)	0.8 (0.5, 1.2)
	MaxHarv	2026 - 2033	3.7	2.9	-0.4 (-1, 0.1)	2.4 (1.9, 3)
	MaxHarv	2034 - 2050	2.2	1.7	-0.3 (-0.6, 0)	1.4 (0.7, 1.9)
10<br>South Savo	BaseHarv	2017 - 2025	7.6	5.7	-4 (-4.9, -2.8)	1.7 (0.8, 2.8)
	BaseHarv	2026 - 2033	8	6.2	-1.3 (-2.9, 0.1)	4.8 (3.5, 6.4)
	BaseHarv	2034 - 2050	5.9	4.6	-0.2 (-1.3, 0.7)	4.4 (2.7, 5.5)
	LowHarv	2017 - 2025	6.2	4.7	-4.2 (-5.1, -3.1)	0.5 (-0.5, 1.6)
	LowHarv	2026 - 2033	4.8	3.6	-2.2 (-3.3, -1.1)	1.4 (0.3, 2.4)
	LowHarv	2034 - 2050	4.8	3.4	-2.2 (-3, -1.2)	1.3 (0.4, 2.5)

	MaxHarv	2017 - 2025	8.3	6.2	-3.8 (-4.7, -2.6)	2.4 (1.6, 3.5)
	MaxHarv	2026 - 2033	9.5	7.4	-0.4 (-1.9, 0.9)	7 (5.6, 8.4)
	MaxHarv	2034 - 2050	4.8	3.7	-0.2 (-1.2, 0.5)	3.5 (1.6, 5.1)
11<br>North Savo	BaseHarv	2017 - 2025	7.7	5.6	-6.1 (-7.3, -4.7)	-0.5 (-1.7, 1)
	BaseHarv	2026 - 2033	8	6	-3.7 (-5, -2.2)	2.3 (1, 3.7)
	BaseHarv	2034 - 2050	7.9	6.1	-2.4 (-3.8, -0.3)	3.6 (2.1, 5.6)
	LowHarv	2017 - 2025	6.3	4.6	-6.2 (-7.3, -4.7)	-1.5 (-2.7, -0.1)
	LowHarv	2026 - 2033	4.8	3.5	-4.4 (-5.8, -3.2)	-0.9 (-2.3, 0.4)
	LowHarv	2034 - 2050	4.8	3.4	-4.2 (-5.1, -3)	-0.8 (-1.8, 0.5)
	MaxHarv	2017 - 2025	8.4	6.1	-5.9 (-7, -4.4)	0.3 (-0.9, 1.7)
	MaxHarv	2026 - 2033	9.6	7.2	-2.9 (-4.3, -1.2)	4.3 (3, 5.8)
	MaxHarv	2034 - 2050	9.1	7.1	-1.1 (-2.8, 0.7)	5.9 (4.6, 7.3)
12<br>North Karelia	BaseHarv	2017 - 2025	6.6	4.7	-6.5 (-7.7, -5.2)	-1.7 (-2.9, -0.4)
	BaseHarv	2026 - 2033	6.8	4.9	-4 (-5.5, -2.6)	1 (-0.6, 2.3)
	BaseHarv	2034 - 2050	6.8	4.9	-3.2 (-4.2, -2.1)	1.7 (0.7, 3)
	LowHarv	2017 - 2025	5.4	3.9	-6.8 (-8.1, -5.5)	-2.9 (-4.2, -1.5)
	LowHarv	2026 - 2033	4.1	2.9	-5.4 (-7, -3.9)	-2.4 (-4.1, -1)
	LowHarv	2034 - 2050	4.1	2.9	-4.9 (-6.1, -3.6)	-2 (-3.3, -0.7)
	MaxHarv	2017 - 2025	7.2	5.1	-6.1 (-7.5, -4.9)	-1 (-2.2, 0.4)
	MaxHarv	2026 - 2033	8.2	5.9	-3.2 (-4.7, -1.8)	2.8 (1.2, 4.1)
	MaxHarv	2034 - 2050	8.1	6	-2.4 (-3.5, -1)	3.6 (2.4, 5)
13<br>Central Finland	BaseHarv	2017 - 2025	7.6	5.6	-6.5 (-7.6, -5)	-0.8 (-2, 0.6)
	BaseHarv	2026 - 2033	8	6.1	-3.9 (-5.6, -1.9)	2.1 (0.4, 4.1)
	BaseHarv	2034 - 2050	7.9	6.1	-2.2 (-4.3, 0.3)	3.9 (1.8, 6.2)
	LowHarv	2017 - 2025	6.3	4.6	-6.5 (-7.7, -5)	-1.8 (-3, -0.6)
	LowHarv	2026 - 2033	4.8	3.5	-4.6 (-5.8, -3.4)	-1 (-2.2, 0.1)
	LowHarv	2034 - 2050	4.8	3.4	-4.2 (-5.2, -3)	-0.8 (-1.8, 0.5)
	MaxHarv	2017 - 2025	8.3	6.2	-6.3 (-7.4, -4.8)	-0.1 (-1.2, 1.2)
	MaxHarv	2026 - 2033	9.6	7.3	-3.2 (-5.1, -0.9)	4.1 (2.4, 6.4)
	MaxHarv	2034 - 2050	8.7	6.8	-1 (-3.1, 0.9)	5.8 (4.3, 7.2)
14<br>South Ostrobothnia	BaseHarv	2017 - 2025	3.8	2.8	-4.7 (-5.6, -3.8)	-1.9 (-2.8, -1)
	BaseHarv	2026 - 2033	3.9	2.9	-3.2 (-4.1, -2.2)	-0.3 (-1.3, 0.7)
	BaseHarv	2034 - 2050	3.9	2.9	-2.4 (-3.2, -1.3)	0.5 (-0.3, 1.5)
	LowHarv	2017 - 2025	3.1	2.3	-4.8 (-5.7, -3.8)	-2.5 (-3.4, -1.6)
	LowHarv	2026 - 2033	2.4	1.7	-3.8 (-4.7, -2.9)	-2.1 (-2.9, -1.2)
	LowHarv	2034 - 2050	2.3	1.7	-3.5 (-4.2, -2.7)	-1.8 (-2.6, -1)
	MaxHarv	2017 - 2025	4.1	3	-4.6 (-5.5, -3.6)	-1.6 (-2.4, -0.6)
	MaxHarv	2026 - 2033	4.7	3.5	-2.7 (-3.7, -1.7)	0.8 (-0.3, 1.8)
	MaxHarv	2034 - 2050	4.7	3.5	-1.9 (-2.7, -0.4)	1.6 (0.8, 3.1)
15<br>Ostrobothnia	BaseHarv	2017 - 2025	2.8	2	-2.9 (-3.3, -2.3)	-0.9 (-1.3, -0.3)
	BaseHarv	2026 - 2033	2.9	2.1	-2 (-2.5, -1.5)	0.1 (-0.4, 0.6)
	BaseHarv	2034 - 2050	2.9	2.1	-1.6 (-2, -1)	0.5 (0.1, 1.1)
	LowHarv	2017 - 2025	2.3	1.7	-2.9 (-3.4, -2.4)	-1.3 (-1.8, -0.8)
	LowHarv	2026 - 2033	1.7	1.2	-2.3 (-2.8, -1.8)	-1.1 (-1.5, -0.5)
	LowHarv	2034 - 2050	1.7	1.2	-2.1 (-2.5, -1.6)	-0.9 (-1.3, -0.4)
	MaxHarv	2017 - 2025	3	2.2	-2.8 (-3.3, -2.2)	-0.6 (-1.1, -0.1)
	MaxHarv	2026 - 2033	3.5	2.6	-1.8 (-2.3, -1.3)	0.8 (0.3, 1.3)
	MaxHarv	2034 - 2050	3.4	2.6	-1.3 (-1.8, -0.5)	1.3 (0.8, 2.1)
16<br>Central Ostrobothnia	BaseHarv	2017 - 2025	1.2	0.9	-1.5 (-1.8, -1.1)	-0.6 (-0.9, -0.2)
	BaseHarv	2026 - 2033	1.3	0.9	-1 (-1.3, -0.5)	0 (-0.4, 0.4)

	BaseHarv	2034 - 2050	1.3	0.9	-0.7 (-1, -0.3)	0.3 (0, 0.6)
	LowHarv	2017 - 2025	1	0.7	-1.5 (-1.9, -1.1)	-0.8 (-1.1, -0.4)
	LowHarv	2026 - 2033	0.8	0.6	-1.2 (-1.6, -0.8)	-0.6 (-1, -0.3)
	LowHarv	2034 - 2050	0.8	0.5	-1.1 (-1.4, -0.7)	-0.5 (-0.8, -0.2)
	MaxHarv	2017 - 2025	1.3	1	-1.4 (-1.8, -1)	-0.5 (-0.8, -0.1)
	MaxHarv	2026 - 2033	1.5	1.1	-0.8 (-1.2, -0.4)	0.3 (0, 0.7)
	MaxHarv	2034 - 2050	1.5	1.1	-0.5 (-0.8, 0)	0.6 (0.3, 1)
17 North Ostrobothnia	BaseHarv	2017 - 2025	7.3	5.3	-9.3 (-11.3, -6.5)	-4 (-5.9, -1.4)
	BaseHarv	2026 - 2033	7.6	5.5	-6.5 (-9.1, -3.7)	-0.9 (-3.5, 1.6)
	BaseHarv	2034 - 2050	7.5	5.5	-4.9 (-7, -2.7)	0.5 (-1.6, 2.7)
	LowHarv	2017 - 2025	6.1	4.4	-9.8 (-11.9, -7)	-5.4 (-7.5, -2.8)
	LowHarv	2026 - 2033	4.6	3.3	-8.2 (-11, -5.7)	-4.9 (-7.7, -2.5)
	LowHarv	2034 - 2050	4.5	3.3	-7.4 (-9.6, -5.3)	-4.2 (-6.3, -2.1)
	MaxHarv	2017 - 2025	8	5.8	-8.9 (-11, -6.2)	-3.1 (-5.1, -0.5)
	MaxHarv	2026 - 2033	9.1	6.7	-5.4 (-8, -2.7)	1.3 (-1.3, 3.9)
	MaxHarv	2034 - 2050	9	6.6	-3.7 (-5.8, -1.1)	2.9 (0.7, 5.5)
18 Kainuu	BaseHarv	2017 - 2025	4	2.9	-6.8 (-8.1, -5.2)	-4 (-5.2, -2.5)
	BaseHarv	2026 - 2033	4.1	3	-5.1 (-6.8, -3.6)	-2.2 (-3.8, -0.7)
	BaseHarv	2034 - 2050	4.1	2.9	-4.3 (-5.4, -2.9)	-1.3 (-2.5, 0)
	LowHarv	2017 - 2025	3.3	2.4	-7.1 (-8.4, -5.5)	-4.7 (-6, -3.2)
	LowHarv	2026 - 2033	2.5	1.8	-6.3 (-7.9, -4.8)	-4.5 (-6.1, -3)
	LowHarv	2034 - 2050	2.5	1.8	-5.7 (-6.9, -4.4)	-4 (-5.2, -2.7)
	MaxHarv	2017 - 2025	4.3	3.1	-6.6 (-7.9, -5)	-3.5 (-4.8, -2)
	MaxHarv	2026 - 2033	5	3.6	-4.5 (-6.1, -2.9)	-0.9 (-2.5, 0.5)
	MaxHarv	2034 - 2050	4.9	3.5	-3.6 (-4.8, -2.3)	-0.1 (-1.3, 1.2)
19 Lapland	BaseHarv	2017 - 2025	5.2	3.8	-16.7 (-20.1, -12.9)	-13 (-16.3, -9.2)
	BaseHarv	2026 - 2033	5.5	3.9	-15.2 (-19.7, -11.1)	-11.3 (-15.9, -7.2)
	BaseHarv	2034 - 2050	5.5	3.9	-13.7 (-17, -10)	-9.8 (-12.9, -6.2)
	LowHarv	2017 - 2025	4.3	3.1	-17.1 (-20.3, -13.3)	-14 (-17.2, -10.3)
	LowHarv	2026 - 2033	3.3	2.4	-16.9 (-21.2, -12.8)	-14.5 (-18.9, -10.5)
	LowHarv	2034 - 2050	3.3	2.3	-16.2 (-19.6, -12.4)	-13.9 (-17.1, -10.2)
	MaxHarv	2017 - 2025	5.7	4.1	-16.6 (-19.9, -12.7)	-12.5 (-15.8, -8.6)
	MaxHarv	2026 - 2033	6.6	4.7	-14.4 (-18.8, -10.2)	-9.7 (-14.2, -5.7)
	MaxHarv	2034 - 2050	6.5	4.7	-12.5 (-15.6, -8.7)	-7.8 (-10.8, -4.2)

Table S2_3. Impact of forest harvesting intensity and climate change scenarios (PREBAS model, RCP4.5 climate change scenario) on aggregated GHG emissions and sinks ($TgCO_2eq\ a^{-1}$) for forested areas of the 18 administrative regions of mainland Finland (see Fig. 2 in main paper). The forest harvesting intensity scenarios (BaseHarv, LowHarv, MaxHarv) are based on the national climate and energy strategy and forestry policies. For forests, emissions are assumed to equal harvested biomass and sinks NEE (net ecosystem exchange). The net emissions of forested areas (NBE) were calculated by adding NEE estimates to the amount of harvested biomass and considering CH_4 and N_2O flux from organic soils. Positive values indicate emissions and negative values sinks. Results for forests are shown as averages for several years to reduce the annual variability caused by changes in climate conditions. The present situation refers to average result for years 2017-2025. The best available estimates of uncertainty (95%) are also shown for NEE and NBE. Corresponding results assuming current climate conditions are shown in Table S2_2. See main paper for details.

Region	Scenario	Period	Harvested volume $Mm^3\ a^{-1}$	Harvested biomass $TgCO_2eq\ a^{-1}$	NEE (95%) $TgCO_2eq\ a^{-1}$	NBE (95%) $TgCO_2eq\ a^{-1}$
01 Uusimaa	BaseHarv	2017 - 2025	4	3	-3.7 (-4.4, -2.8)	-0.8 (-1.4, 0)
	BaseHarv	2026 - 2033	4.3	3.2	-3.1 (-3.8, -2.1)	0.1 (-0.6, 1.1)
	BaseHarv	2034 - 2050	4.2	3.3	-2.6 (-3.5, -1.5)	0.7 (-0.2, 1.7)
	LowHarv	2017 - 2025	3.3	2.4	-3.8 (-4.4, -2.9)	-1.4 (-2, -0.6)
	LowHarv	2026 - 2033	2.6	1.9	-3.5 (-4, -2.8)	-1.6 (-2, -0.8)
	LowHarv	2034 - 2050	2.5	1.8	-3.6 (-4.2, -2.9)	-1.8 (-2.4, -1.1)
	MaxHarv	2017 - 2025	4.4	3.2	-3.7 (-4.3, -2.7)	-0.4 (-1.1, 0.3)
	MaxHarv	2026 - 2033	5.1	3.9	-2.7 (-3.5, -1.8)	1.2 (0.4, 2.1)
	MaxHarv	2034 - 2050	4.8	3.7	-2 (-2.9, -1.2)	1.7 (1, 2.4)
02 Southwest Finland	BaseHarv	2017 - 2025	3.6	2.6	-4.2 (-4.9, -3.2)	-1.6 (-2.2, -0.7)
	BaseHarv	2026 - 2033	3.7	2.8	-3.6 (-4.3, -2.9)	-0.9 (-1.5, -0.1)
	BaseHarv	2034 - 2050	3.7	2.7	-3.4 (-4.1, -2.7)	-0.7 (-1.4, 0.2)
	LowHarv	2017 - 2025	2.9	2.1	-4.2 (-4.9, -3.3)	-2.1 (-2.8, -1.3)
	LowHarv	2026 - 2033	2.2	1.6	-4.1 (-4.6, -3.3)	-2.5 (-3, -1.7)
	LowHarv	2034 - 2050	2.2	1.6	-4.4 (-5.1, -3.4)	-2.8 (-3.6, -1.8)
	MaxHarv	2017 - 2025	3.9	2.8	-4.1 (-4.8, -3.2)	-1.3 (-1.9, -0.5)
	MaxHarv	2026 - 2033	4.5	3.3	-3.3 (-3.9, -2.6)	0 (-0.6, 0.9)
	MaxHarv	2034 - 2050	4.4	3.3	-3 (-3.7, -2.2)	0.3 (-0.4, 1.2)
04 Satakunta	BaseHarv	2017 - 2025	3.1	2.3	-3 (-3.5, -2.3)	-0.8 (-1.3, -0.1)
	BaseHarv	2026 - 2033	3.2	2.4	-2.4 (-2.9, -1.8)	0 (-0.6, 0.5)
	BaseHarv	2034 - 2050	3.2	2.3	-2.2 (-2.8, -1.7)	0.1 (-0.5, 0.8)
	LowHarv	2017 - 2025	2.5	1.9	-3.1 (-3.6, -2.4)	-1.2 (-1.7, -0.5)
	LowHarv	2026 - 2033	1.9	1.4	-2.8 (-3.4, -2.3)	-1.5 (-1.9, -0.9)
	LowHarv	2034 - 2050	1.9	1.3	-3.1 (-3.8, -2.4)	-1.8 (-2.4, -1)
	MaxHarv	2017 - 2025	3.4	2.5	-2.9 (-3.5, -2.2)	-0.5 (-1, 0.2)
	MaxHarv	2026 - 2033	3.8	2.9	-2 (-2.6, -1.5)	0.8 (0.3, 1.4)
	MaxHarv	2034 - 2050	3.8	2.8	-1.9 (-2.4, -1.2)	1 (0.3, 1.7)
05 Kanta-Häme	BaseHarv	2017 - 2025	2.7	2	-1.8 (-2.2, -1.4)	0.2 (-0.1, 0.6)
	BaseHarv	2026 - 2033	2.8	2.1	-1.4 (-1.7, -0.9)	0.7 (0.4, 1.3)
	BaseHarv	2034 - 2050	2.7	2.1	-1.1 (-1.5, -0.7)	1 (0.6, 1.4)
	LowHarv	2017 - 2025	2.2	1.6	-1.8 (-2.2, -1.4)	-0.2 (-0.5, 0.3)
	LowHarv	2026 - 2033	1.7	1.2	-1.6 (-2, -1.2)	-0.4 (-0.8, 0.1)
	LowHarv	2034 - 2050	1.7	1.2	-1.9 (-2.3, -1.4)	-0.7 (-1.1, -0.2)
	MaxHarv	2017 - 2025	2.9	2.1	-1.7 (-2.1, -1.3)	0.5 (0.1, 0.9)
	MaxHarv	2026 - 2033	3.4	2.6	-1.1 (-1.4, -0.6)	1.5 (1.1, 2)
	MaxHarv	2034 - 2050	2.7	2.1	-0.9 (-1.2, -0.6)	1.2 (0.5, 1.6)
06 Pirkanmaa	BaseHarv	2017 - 2025	6.3	4.6	-5.7 (-6.8, -4.6)	-1.1 (-2.3, 0.2)
	BaseHarv	2026 - 2033	6.5	4.9	-4.8 (-5.8, -3.4)	0.2 (-0.9, 1.5)

	BaseHarv	2034 - 2050	6.5	4.9	-4.1 (-5.3, -2.5)	0.8 (-0.4, 2.4)
	LowHarv	2017 - 2025	5.2	3.8	-5.7 (-6.9, -4.5)	-2 (-3.1, -0.7)
	LowHarv	2026 - 2033	3.9	2.8	-5.3 (-6.3, -4.2)	-2.5 (-3.4, -1.3)
	LowHarv	2034 - 2050	3.9	2.8	-5.7 (-6.8, -4.5)	-2.9 (-4, -1.7)
	MaxHarv	2017 - 2025	6.8	5	-5.6 (-6.7, -4.5)	-0.6 (-1.6, 0.7)
	MaxHarv	2026 - 2033	7.8	5.9	-4.1 (-5.1, -2.8)	1.8 (0.7, 3.1)
	MaxHarv	2034 - 2050	7.7	5.9	-3.3 (-4.6, -1.6)	2.6 (1.2, 4.1)
07 Päijät-Häme	BaseHarv	2017 - 2025	2.9	2.1	-2.3 (-2.8, -1.8)	-0.2 (-0.7, 0.3)
	BaseHarv	2026 - 2033	3	2.2	-1.9 (-2.4, -1.3)	0.3 (-0.1, 1.1)
	BaseHarv	2034 - 2050	2.9	2.2	-1.7 (-2.1, -1.1)	0.6 (0.1, 1.2)
	LowHarv	2017 - 2025	2.4	1.7	-2.3 (-2.8, -1.8)	-0.6 (-1.1, 0)
	LowHarv	2026 - 2033	1.8	1.3	-2.2 (-2.7, -1.6)	-0.9 (-1.4, -0.3)
	LowHarv	2034 - 2050	1.8	1.3	-2.4 (-2.8, -1.8)	-1.1 (-1.5, -0.6)
	MaxHarv	2017 - 2025	3.1	2.3	-2.2 (-2.7, -1.7)	0 (-0.4, 0.6)
	MaxHarv	2026 - 2033	3.6	2.7	-1.7 (-2.1, -1)	1 (0.6, 1.8)
	MaxHarv	2034 - 2050	3.5	2.7	-1.3 (-1.8, -0.8)	1.3 (0.9, 1.8)
08 Kymenlaakso	BaseHarv	2017 - 2025	2.3	1.7	-1.5 (-1.9, -1.1)	0.3 (-0.1, 0.6)
	BaseHarv	2026 - 2033	2.5	1.9	-1 (-1.5, -0.5)	0.9 (0.4, 1.4)
	BaseHarv	2034 - 2050	2.4	1.8	-0.8 (-1.2, -0.4)	1 (0.6, 1.4)
	LowHarv	2017 - 2025	1.9	1.4	-1.5 (-1.9, -1.2)	-0.1 (-0.5, 0.3)
	LowHarv	2026 - 2033	1.5	1.1	-1.3 (-1.7, -0.8)	-0.2 (-0.6, 0.4)
	LowHarv	2034 - 2050	1.5	1.1	-1.6 (-1.9, -1.2)	-0.5 (-0.9, -0.1)
	MaxHarv	2017 - 2025	2.5	1.9	-1.4 (-1.8, -1.1)	0.5 (0.1, 0.8)
	MaxHarv	2026 - 2033	3	2.3	-0.7 (-1.2, -0.2)	1.6 (1.2, 2.1)
	MaxHarv	2034 - 2050	2.1	1.6	-0.7 (-1, -0.4)	0.9 (0.2, 1.5)
09 South Karelia	BaseHarv	2017 - 2025	2.9	2.2	-1.8 (-2.3, -1.4)	0.4 (-0.1, 0.8)
	BaseHarv	2026 - 2033	3.1	2.4	-1.3 (-1.8, -0.6)	1.1 (0.5, 1.6)
	BaseHarv	2034 - 2050	2.9	2.3	-1 (-1.6, -0.5)	1.3 (0.8, 1.8)
	LowHarv	2017 - 2025	2.4	1.8	-1.9 (-2.4, -1.4)	-0.1 (-0.6, 0.4)
	LowHarv	2026 - 2033	1.9	1.4	-1.7 (-2.2, -1.1)	-0.3 (-0.8, 0.4)
	LowHarv	2034 - 2050	1.8	1.3	-2 (-2.4, -1.4)	-0.7 (-1.1, -0.1)
	MaxHarv	2017 - 2025	3.1	2.4	-1.7 (-2.2, -1.3)	0.7 (0.2, 1.2)
	MaxHarv	2026 - 2033	3.7	2.8	-0.9 (-1.5, -0.3)	1.9 (1.4, 2.4)
	MaxHarv	2034 - 2050	2.7	2.1	-0.8 (-1.2, -0.5)	1.2 (0.4, 1.8)
10 South Savo	BaseHarv	2017 - 2025	7.6	5.7	-4.4 (-5.5, -3.4)	1.3 (0.3, 2.4)
	BaseHarv	2026 - 2033	8	6.2	-2.7 (-4.1, -1.3)	3.5 (2.1, 4.8)
	BaseHarv	2034 - 2050	7.1	5.5	-1.8 (-3.2, -0.6)	3.7 (2.2, 4.8)
	LowHarv	2017 - 2025	6.2	4.7	-4.6 (-5.6, -3.5)	0.1 (-1, 1.2)
	LowHarv	2026 - 2033	4.8	3.6	-3.7 (-4.8, -2.5)	-0.1 (-1.2, 1.2)
	LowHarv	2034 - 2050	4.8	3.4	-4.4 (-5.5, -2.9)	-1 (-2.1, 0.6)
	MaxHarv	2017 - 2025	8.3	6.2	-4.1 (-5.2, -3.2)	2.1 (1, 3.2)
	MaxHarv	2026 - 2033	9.6	7.4	-1.7 (-3, -0.4)	5.7 (4.4, 6.9)
	MaxHarv	2034 - 2050	5.9	4.6	-1.6 (-2.6, -0.7)	3 (0.9, 4.6)
11 North Savo	BaseHarv	2017 - 2025	7.7	5.7	-6.8 (-8.2, -5.4)	-1.1 (-2.6, 0.4)
	BaseHarv	2026 - 2033	8	6	-5.3 (-6.7, -3.5)	0.7 (-0.6, 2.6)
	BaseHarv	2034 - 2050	7.9	6	-4.6 (-6.2, -2.7)	1.4 (-0.3, 3.4)
	LowHarv	2017 - 2025	6.3	4.7	-6.9 (-8.4, -5.5)	-2.2 (-3.8, -0.5)
	LowHarv	2026 - 2033	4.8	3.5	-6.4 (-7.6, -4.6)	-2.9 (-4, -1.1)
	LowHarv	2034 - 2050	4.8	3.4	-6.9 (-8.5, -5.1)	-3.5 (-5.1, -1.7)
	MaxHarv	2017 - 2025	8.4	6.2	-6.5 (-8, -5.1)	-0.4 (-1.9, 1.3)

	MaxHarv	2026 - 2033	9.6	7.2	-4.5 (-5.8, -2.5)	2.8 (1.5, 4.7)
	MaxHarv	2034 - 2050	9.4	7.2	-3.5 (-5.4, -1.3)	3.8 (1.7, 5.7)
12 North Karelia	BaseHarv	2017 - 2025	6.6	4.8	-7.4 (-9.1, -5.9)	-2.6 (-4.3, -1.1)
	BaseHarv	2026 - 2033	6.8	5	-6.2 (-7.5, -4.6)	-1.2 (-2.6, 0.5)
	BaseHarv	2034 - 2050	6.8	4.9	-5.9 (-7.7, -4.1)	-1 (-2.9, 1.1)
	LowHarv	2017 - 2025	5.4	3.9	-7.7 (-9.5, -6.2)	-3.8 (-5.5, -2.2)
	LowHarv	2026 - 2033	4.1	3	-7.9 (-9.2, -6.1)	-4.9 (-6.3, -3.1)
	LowHarv	2034 - 2050	4.1	2.9	-8.2 (-9.9, -6.4)	-5.3 (-6.9, -3.4)
	MaxHarv	2017 - 2025	7.2	5.2	-7.1 (-8.8, -5.6)	-1.9 (-3.6, -0.3)
	MaxHarv	2026 - 2033	8.2	6	-5.2 (-6.5, -3.6)	0.7 (-0.7, 2.4)
	MaxHarv	2034 - 2050	8.1	5.9	-5 (-6.9, -3.2)	0.8 (-1.2, 3)
13 Central Finland	BaseHarv	2017 - 2025	7.6	5.7	-7.2 (-8.6, -5.9)	-1.5 (-3, 0)
	BaseHarv	2026 - 2033	8	6.1	-5.7 (-7.5, -3.7)	0.3 (-1.4, 2.2)
	BaseHarv	2034 - 2050	8	6.1	-4.6 (-6.6, -2)	1.4 (-0.6, 3.9)
	LowHarv	2017 - 2025	6.3	4.7	-7.2 (-8.7, -5.9)	-2.6 (-4, -1)
	LowHarv	2026 - 2033	4.8	3.6	-6.6 (-7.8, -5.1)	-3.1 (-4.3, -1.7)
	LowHarv	2034 - 2050	4.8	3.4	-7 (-8.6, -5.5)	-3.6 (-5.1, -1.9)
	MaxHarv	2017 - 2025	8.3	6.2	-7 (-8.4, -5.8)	-0.8 (-2.2, 0.7)
	MaxHarv	2026 - 2033	9.6	7.3	-4.9 (-6.7, -2.9)	2.4 (0.7, 4.3)
	MaxHarv	2034 - 2050	9.4	7.3	-3.4 (-5.7, -0.9)	3.9 (1.6, 5.8)
14 South Ostrobothnia	BaseHarv	2017 - 2025	3.8	2.8	-5.4 (-6.5, -4.4)	-2.6 (-3.7, -1.6)
	BaseHarv	2026 - 2033	3.9	2.9	-4.5 (-5.6, -3.4)	-1.6 (-2.6, -0.5)
	BaseHarv	2034 - 2050	3.9	2.9	-4.3 (-5.5, -3)	-1.5 (-2.7, -0.2)
	LowHarv	2017 - 2025	3.1	2.3	-5.5 (-6.5, -4.5)	-3.2 (-4.3, -2.2)
	LowHarv	2026 - 2033	2.4	1.7	-5.3 (-6.2, -4.5)	-3.6 (-4.5, -2.7)
	LowHarv	2034 - 2050	2.3	1.7	-5.7 (-6.8, -4.8)	-4 (-5.2, -3.1)
	MaxHarv	2017 - 2025	4.1	3	-5.3 (-6.3, -4.3)	-2.3 (-3.3, -1.2)
	MaxHarv	2026 - 2033	4.7	3.5	-4 (-4.9, -2.9)	-0.5 (-1.4, 0.5)
	MaxHarv	2034 - 2050	4.7	3.4	-3.8 (-5, -2.3)	-0.3 (-1.6, 1.3)
15 Ostrobothnia	BaseHarv	2017 - 2025	2.8	2.1	-3.2 (-3.7, -2.6)	-1.1 (-1.6, -0.5)
	BaseHarv	2026 - 2033	2.9	2.1	-2.6 (-3.1, -2)	-0.5 (-0.9, 0.1)
	BaseHarv	2034 - 2050	2.9	2.1	-2.5 (-3.1, -1.8)	-0.4 (-1.1, 0.3)
	LowHarv	2017 - 2025	2.3	1.7	-3.2 (-3.7, -2.6)	-1.5 (-2, -0.9)
	LowHarv	2026 - 2033	1.7	1.3	-3 (-3.5, -2.5)	-1.8 (-2.3, -1.2)
	LowHarv	2034 - 2050	1.7	1.2	-3.2 (-3.9, -2.7)	-2 (-2.6, -1.4)
	MaxHarv	2017 - 2025	3	2.2	-3.1 (-3.6, -2.5)	-0.9 (-1.3, -0.3)
	MaxHarv	2026 - 2033	3.5	2.6	-2.3 (-2.8, -1.8)	0.2 (-0.2, 0.8)
	MaxHarv	2034 - 2050	3.4	2.5	-2.2 (-2.8, -1.3)	0.4 (-0.3, 1.3)
16 Central Ostrobothnia	BaseHarv	2017 - 2025	1.2	0.9	-1.8 (-2.2, -1.4)	-0.9 (-1.3, -0.6)
	BaseHarv	2026 - 2033	1.3	0.9	-1.5 (-1.9, -1.1)	-0.6 (-1, -0.2)
	BaseHarv	2034 - 2050	1.3	0.9	-1.5 (-2, -1)	-0.6 (-1.1, -0.1)
	LowHarv	2017 - 2025	1	0.7	-1.9 (-2.3, -1.5)	-1.2 (-1.5, -0.8)
	LowHarv	2026 - 2033	0.8	0.6	-1.8 (-2.2, -1.4)	-1.3 (-1.6, -0.9)
	LowHarv	2034 - 2050	0.8	0.5	-1.9 (-2.5, -1.5)	-1.4 (-1.9, -1)
	MaxHarv	2017 - 2025	1.3	1	-1.8 (-2.2, -1.4)	-0.8 (-1.2, -0.4)
	MaxHarv	2026 - 2033	1.5	1.1	-1.3 (-1.7, -1)	-0.2 (-0.6, 0.2)
	MaxHarv	2034 - 2050	1.5	1.1	-1.3 (-1.8, -0.8)	-0.2 (-0.7, 0.3)
17 North Ostrobothnia	BaseHarv	2017 - 2025	7.3	5.4	-12.1 (-15.8, -8.9)	-6.8 (-10.2, -3.7)
	BaseHarv	2026 - 2033	7.6	5.6	-11.1 (-14.1, -7.7)	-5.5 (-8.4, -2.4)
	BaseHarv	2034 - 2050	7.5	5.5	-10.9 (-14.8, -7.5)	-5.4 (-9.4, -1.9)

	LowHarv	2017 - 2025	6.1	4.4	-12.6 (-16.5, -9.5)	-8.2 (-11.9, -5.2)
	LowHarv	2026 - 2033	4.6	3.3	-13.1 (-15.7, -10.1)	-9.8 (-12.3, -6.9)
	LowHarv	2034 - 2050	4.5	3.3	-14.1 (-17.9, -10.8)	-10.8 (-14.7, -7.4)
	MaxHarv	2017 - 2025	8	5.8	-11.8 (-15.4, -8.6)	-5.9 (-9.4, -2.9)
	MaxHarv	2026 - 2033	9.1	6.7	-9.8 (-12.8, -6.4)	-3.1 (-5.9, -0.1)
	MaxHarv	2034 - 2050	9.1	6.6	-9.6 (-13.6, -5.7)	-3 (-7.2, 0.9)
18 Kainuu	BaseHarv	2017 - 2025	4	2.9	-8.6 (-10.7, -6.7)	-5.7 (-7.8, -3.9)
	BaseHarv	2026 - 2033	4.1	3	-8.2 (-9.9, -6.5)	-5.2 (-6.8, -3.6)
	BaseHarv	2034 - 2050	4.1	3	-8.1 (-10.4, -5.9)	-5.1 (-7.5, -2.9)
	LowHarv	2017 - 2025	3.3	2.4	-8.8 (-11.1, -6.9)	-6.5 (-8.6, -4.7)
	LowHarv	2026 - 2033	2.5	1.8	-9.5 (-11, -7.8)	-7.7 (-9.2, -6)
	LowHarv	2034 - 2050	2.5	1.8	-9.8 (-12, -7.7)	-8 (-10.3, -5.8)
	MaxHarv	2017 - 2025	4.3	3.2	-8.4 (-10.5, -6.5)	-5.2 (-7.4, -3.4)
	MaxHarv	2026 - 2033	5	3.6	-7.5 (-8.9, -5.9)	-3.9 (-5.2, -2.3)
	MaxHarv	2034 - 2050	4.9	3.5	-7.4 (-9.8, -5.1)	-3.8 (-6.3, -1.7)
19 Lapland	BaseHarv	2017 - 2025	5.2	3.8	-21 (-27, -16.2)	-17.3 (-23.3, -12.3)
	BaseHarv	2026 - 2033	5.5	3.9	-24 (-28.1, -18.9)	-20.1 (-24, -15)
	BaseHarv	2034 - 2050	5.5	3.9	-27.2 (-34.7, -19.7)	-23.3 (-30.8, -15.8)
	LowHarv	2017 - 2025	4.3	3.1	-21.4 (-27.2, -16.4)	-18.3 (-24.2, -13.4)
	LowHarv	2026 - 2033	3.3	2.4	-26 (-30.3, -20.8)	-23.6 (-27.9, -18.5)
	LowHarv	2034 - 2050	3.3	2.4	-30.2 (-37.5, -22.6)	-27.8 (-35.2, -20.3)
	MaxHarv	2017 - 2025	5.7	4.1	-20.8 (-26.7, -15.9)	-16.7 (-22.7, -11.8)
	MaxHarv	2026 - 2033	6.6	4.7	-22.9 (-26.9, -18)	-18.2 (-22.2, -13.3)
	MaxHarv	2034 - 2050	6.6	4.7	-25.9 (-33.4, -18.4)	-21.2 (-28.7, -13.8)

Table S3_1. Area (km^2), forest harvesting scenario, period, volume growth (Mm^3 a^{-1}), C storages in trees+ground vegetation and soils (TgC), harvested volume (Mm^3 a^{-1}), NEE ($TgCO_2eq$ a^{-1}), climate regulation service net C sequestration (NBE) ($TgCO_2eq$ a^{-1}), and climate regulation service C storage (TgC) of forested areas of Finland, for two levels of protected areas (currently protected areas, assuming an increase to 10% strictly protected area), and time periods. PREBAS model simulations have been made assuming the RCP4.5 climate change scenario. Results for forests are shown as averages for several years to reduce the annual variability caused by changes in climate conditions. Negative NBE values indicate sinks and positive values sources. Uncertainty estimates (95%) are shown for the NBE and C storage values. Results are shown separately for the protected areas and the whole forested area (protected + managed area) of Finland. For the protected areas, the NoHarv scenario is assumed. For the whole area, the results of three harvesting intensity scenarios (BaseHarv, LowHarv and MaxHarv) for the forested areas outside the currently strictly protected areas are shown. These harvesting intensity scenarios indicate the implications of intensive vs. conservation-oriented forestry for the managed forest land with respect to key C variables. The climate regulation services net C sequestration (NBE) and C storage are calculated according to SEEA definitions. Corresponding results assuming current climate conditions are shown in Table 3 in the main paper. See main paper for details.

	Area km^2	Scenario	Period	Volume growth Mm^3 a^{-1}	C storage above ground TgC	C storage soils TgC	Harv. volume Mm^3 a^{-1}	NEE $TgCO_2eq$ a^{-1}	Climate serv. net C sequestration (NBE) (95%) $TgCO_2eq$ a^{-1}	Climate serv. C stock (95%) TgC
Currently protected	16428	NoHarv	2017 - 2025	5.7	55.2	176.3	0	-7.1	-7.1 (-8.5, -5.8)	231.5 (216.8, 242.8)
Currently protected	16428	NoHarv	2034 - 2050	7.5	94	177.8	0	-9.4	-9.4 (-11.3, -7.7)	271.8 (255.5, 286.6)
10% target	27053	NoHarv	2017 - 2025	13.8	132.7	245.4	0	-16.2	-16.2 (-18.4, -13.4)	378.1 (355.9, 394.9)
10% target	27053	NoHarv	2034 - 2050	15.1	204.7	247.2	0	-17.5	-17.5 (-20.2, -14.7)	451.9 (431.3, 472.3)
Whole region	211130	BaseHarv	2017 - 2025	122.7	950	2617	81.5	-102	-42 (-61.9, -21.7)	3567 (3382.4, 3717.9)
Whole region	211130	BaseHarv	2034 - 2050	114.1	1038.3	2572.8	83.5	-88.1	-25.8 (-51.4, -1.2)	3611.1 (3366.3, 3819.6)
Whole region	211130	LowHarv	2017 - 2025	123.2	958.9	2617.1	67	-104.1	-54.9 (-75, -36.5)	3576 (3390.7, 3727)
Whole region	211130	LowHarv	2034 - 2050	130.6	1269.2	2603	50.9	-115.9	-79.6 (-102.8, -57.9)	3872.2 (3700.9, 4040.1)
Whole region	211130	MaxHarv	2017 - 2025	122.3	937.2	2613.1	88.7	-99.1	-33.8 (-53.8, -13.9)	3550.4 (3367.6, 3698.6)
Whole region	211130	MaxHarv	2034 - 2050	107.9	900.5	2555.1	94.8	-77.4	-6.2 (-29.6, 16.5)	3455.6 (3226.7, 3667)

Table S3_2. Area (km²), forest harvesting scenario, period, volume growth (Mm³ a⁻¹), C storages in trees+ground vegetation and soils (TgC), harvested volume (Mm³ a⁻¹), NEE (TgCO₂eq a⁻¹), climate regulation service net sequestration (NBE) (TgCO₂eq a⁻¹), and climate regulation service C storage (TgC) for forested areas of the 18 administrative regions of mainland Finland (see Fig. 2 in main paper), for two levels of protected areas (currently protected areas, assuming an increase to 10% strictly protected area), and time periods. PREBAS model simulations have been made assuming current climate conditions. Results for forests are shown as averages for several years to reduce the annual variability caused by changes in climate conditions. Negative NBE values indicate sinks and positive values sources. Uncertainty estimates (95%) are shown for the NBE and C storage values. Results are shown separately for the protected areas and the whole forested area (protected + managed area) of Finland. For the protected areas, the NoHarv scenario is assumed. For the whole area, the results of three harvesting intensity scenarios (BaseHarv, LowHarv and MaxHarv) for the forested areas outside the currently strictly protected areas are shown. These harvesting intensity scenarios indicate the implications of intensive vs. conservation-oriented forestry for the managed forest land with respect to key C variables. The climate regulation services net C sequestration (NBE) and C storage are calculated according to SEEA definitions. Corresponding results assuming the RCP 4.5 climate change scenario are shown in Table S3_3. See main paper for details.

Region		Area km2	Scenario	Period	Volume growth $Mm^3\ a^{-1}$	C storage above ground TgC	C storage soils TgC	Harvested volume $Mm^3\ a^{-1}$	NEE $TgCO_2eq\ a^{-1}$	Climate serv. net C sequestration (NBE) (95%) $TgCO_2eq\ a^{-1}$	Climate serv. C storage (95%) TgC
01 Uusimaa	Currently protected	250	NoHarv	2017 - 2025	0.2	1.8	1.5	0	-0.2	-0.2 (-0.2, -0.2)	3.2 (2.9, 3.4)
	Currently protected	250	NoHarv	2034 - 2050	0.2	2.4	1.5	0	-0.2	-0.2 (-0.2, -0.1)	3.9 (3.7, 4.1)
	10% target	680	NoHarv	2017 - 2025	0.5	4.9	3	0	-0.6	-0.6 (-0.6, -0.5)	8 (7.3, 8.5)
	10% target	680	NoHarv	2034 - 2050	0.4	6.7	3	0	-0.5	-0.5 (-0.5, -0.4)	9.7 (9.1, 10.2)
	Whole region	5761	BaseHarv	2017 - 2025	3.8	32.1	30.4	4	-3.6	-0.6 (-1.1, 0)	62.5 (56, 67)
	Whole region	5761	BaseHarv	2034 - 2050	2.3	23.1	28.3	4.2	-1.5	1.7 (0.8, 2.6)	51.4 (41.9, 58.7)
	Whole region	5761	LowHarv	2017 - 2025	3.9	32.5	30.4	3.3	-3.6	-1.2 (-1.7, -0.6)	62.9 (56.5, 67.2)
	Whole region	5761	LowHarv	2034 - 2050	3.1	32.7	28.8	2.5	-2.5	-0.7 (-1.1, -0.1)	61.5 (55.2, 65.5)
	Whole region	5761	MaxHarv	2017 - 2025	3.8	31.6	30.3	4.4	-3.5	-0.3 (-0.8, 0.3)	61.9 (55.4, 66.2)
	Whole region	5761	MaxHarv	2034 - 2050	2	16.9	27.6	4.2	-1.1	2.2 (1.5, 2.7)	44.5 (37.8, 51)
02 Southwest Finland	Currently protected	216	NoHarv	2017 - 2025	0.1	1.3	3.4	0	-0.2	-0.2 (-0.2, -0.1)	4.7 (4.4, 4.9)
	Currently protected	216	NoHarv	2034 - 2050	0.1	1.9	3.4	0	-0.1	-0.1 (-0.2, -0.1)	5.4 (5.1, 5.6)
	10% target	754	NoHarv	2017 - 2025	0.5	4.9	8.8	0	-0.6	-0.6 (-0.7, -0.5)	13.7 (12.8, 14.4)
	10% target	754	NoHarv	2034 - 2050	0.4	7.1	8.8	0	-0.5	-0.5 (-0.6, -0.4)	15.9 (15.2, 16.5)
	Whole region	6394	BaseHarv	2017 - 2025	4.1	33.5	37.4	3.6	-4	-1.4 (-2.1, -0.7)	70.8 (64.9, 75.3)
	Whole region	6394	BaseHarv	2034 - 2050	2.9	30.7	35.3	3.7	-2.3	0.5 (-0.1, 1.5)	66 (57.4, 70.8)
	Whole region	6394	LowHarv	2017 - 2025	4.1	33.8	37.4	2.9	-4.1	-1.9 (-2.5, -1.2)	71.1 (65.1, 75.6)
	Whole region	6394	LowHarv	2034 - 2050	3.4	38.6	36.2	2.2	-3.1	-1.5 (-2, -1)	74.8 (69.5, 78.7)
	Whole region	6394	MaxHarv	2017 - 2025	4.1	33	37.2	3.9	-4	-1.1 (-1.7, -0.4)	70.2 (64.3, 74.7)
	Whole region	6394	MaxHarv	2034 - 2050	2.6	25.1	34.6	4.4	-1.8	1.5 (0.8, 2.3)	59.7 (51.1, 65)
04 Satakunta	Currently protected	120	NoHarv	2017 - 2025	0.1	0.7	1	0	-0.1	-0.1 (-0.1, -0.1)	1.7 (1.6, 1.8)
	Currently protected	120	NoHarv	2034 - 2050	0.1	1.1	1	0	-0.1	-0.1 (-0.1, -0.1)	2.1 (1.9, 2.2)
	10% target	541	NoHarv	2017 - 2025	0.4	3.8	2.9	0	-0.4	-0.4 (-0.5, -0.4)	6.7 (6.1, 7.1)
	10% target	541	NoHarv	2034 - 2050	0.3	5.2	2.9	0	-0.3	-0.3 (-0.4, -0.3)	8.1 (7.6, 8.4)
	Whole region	5294	BaseHarv	2017 - 2025	3.5	28.5	57.2	3.1	-2.8	-0.6 (-1, 0)	85.7 (80.3, 90.8)

	Whole region	5294	BaseHarv	2034 - 2050	2.4	23.4	54.9	3.2	-1.3	1.1 (0.6, 1.8)	78.2 (71.4, 83.4)
	Whole region	5294	LowHarv	2017 - 2025	3.5	28.8	57.2	2.5	-2.9	-1 (-1.4, -0.5)	86 (80.6, 91.1)
	Whole region	5294	LowHarv	2034 - 2050	2.8	30.5	55.7	1.9	-2.1	-0.7 (-1.1, -0.2)	86.2 (81.2, 90.5)
	Whole region	5294	MaxHarv	2017 - 2025	3.5	28	57	3.4	-2.7	-0.3 (-0.7, 0.2)	85.1 (79.7, 90.1)
	Whole region	5294	MaxHarv	2034 - 2050	2.1	18.2	54.1	3.7	-0.9	1.9 (1.4, 2.5)	72.3 (65.4, 77.7)
05 Kanta-Häme	Currently protected	86	NoHarv	2017 - 2025	0.1	0.7	0.5	0	-0.1	-0.1 (-0.1, -0.1)	1.1 (1, 1.2)
	Currently protected	86	NoHarv	2034 - 2050	0.1	0.9	0.5	0	-0.1	-0.1 (-0.1, 0)	1.4 (1.3, 1.4)
	10% target	394	NoHarv	2017 - 2025	0.3	3.2	2.2	0	-0.3	-0.3 (-0.4, -0.3)	5.5 (5, 5.9)
	10% target	394	NoHarv	2034 - 2050	0.2	4	2.3	0	-0.3	-0.3 (-0.3, -0.2)	6.3 (5.8, 6.5)
	Whole region	3554	BaseHarv	2017 - 2025	2.4	20.5	31.5	2.7	-1.7	0.3 (0, 0.6)	52 (47, 55.4)
	Whole region	3554	BaseHarv	2034 - 2050	1.4	11.6	29.5	2.6	-0.5	1.5 (1.1, 1.8)	41.1 (35.5, 45.6)
	Whole region	3554	LowHarv	2017 - 2025	2.4	20.6	31.5	2.2	-1.7	-0.1 (-0.4, 0.3)	52.1 (47.3, 55.5)
	Whole region	3554	LowHarv	2034 - 2050	2	18.5	30.1	1.7	-1.2	0 (-0.3, 0.4)	48.7 (44.7, 51.1)
	Whole region	3554	MaxHarv	2017 - 2025	2.4	20	31.4	2.9	-1.6	0.5 (0.2, 0.8)	51.5 (46.6, 54.9)
	Whole region	3554	MaxHarv	2034 - 2050	1.3	8.6	28.9	2.3	-0.4	1.4 (0.7, 1.8)	37.5 (34.1, 40.5)
06 Pirkanmaa	Currently protected	215	NoHarv	2017 - 2025	0.1	1.7	1.2	0	-0.2	-0.2 (-0.2, -0.1)	2.9 (2.6, 3.1)
	Currently protected	215	NoHarv	2034 - 2050	0.1	2.2	1.2	0	-0.1	-0.1 (-0.1, -0.1)	3.4 (3.2, 3.6)
	10% target	1077	NoHarv	2017 - 2025	0.8	8.7	5.4	0	-0.9	-0.9 (-1, -0.8)	14.1 (12.9, 15)
	10% target	1077	NoHarv	2034 - 2050	0.6	10.9	5.4	0	-0.7	-0.7 (-0.7, -0.6)	16.3 (15.2, 17)
	Whole region	10031	BaseHarv	2017 - 2025	6.6	58.5	92.2	6.3	-5.3	-0.7 (-1.5, 0.3)	150.7 (140.8, 159.4)
	Whole region	10031	BaseHarv	2034 - 2050	4.3	45.2	87.6	6.5	-2.4	2.6 (1.3, 4.3)	132.8 (117.6, 142.2)
	Whole region	10031	LowHarv	2017 - 2025	6.6	59	92.2	5.2	-5.3	-1.6 (-2.4, -0.5)	151.1 (141.4, 159.7)
	Whole region	10031	LowHarv	2034 - 2050	5.3	58.7	88.7	3.9	-3.7	-0.9 (-1.7, 0)	147.5 (138.7, 155)
	Whole region	10031	MaxHarv	2017 - 2025	6.6	57.5	92	6.8	-5.2	-0.2 (-1, 0.9)	149.5 (139.8, 158)
	Whole region	10031	MaxHarv	2034 - 2050	3.8	34.4	86.2	7.5	-1.5	4.3 (3.2, 5.2)	120.5 (107.6, 130.1)
07 Päijät-Häme	Currently protected	76	NoHarv	2017 - 2025	0.1	0.6	1.1	0	-0.1	-0.1 (-0.1, -0.1)	1.7 (1.6, 1.8)
	Currently protected	76	NoHarv	2034 - 2050	0	0.8	1.1	0	0	0 (-0.1, 0)	1.9 (1.8, 2)
	10% target	480	NoHarv	2017 - 2025	0.4	4	4.3	0	-0.4	-0.4 (-0.4, -0.3)	8.3 (7.7, 8.7)
	10% target	480	NoHarv	2034 - 2050	0.3	4.9	4.3	0	-0.3	-0.3 (-0.3, -0.3)	9.2 (8.6, 9.5)
	Whole region	4202	BaseHarv	2017 - 2025	2.8	24.8	26.6	2.9	-2.2	-0.1 (-0.5, 0.4)	51.5 (46.6, 55)
	Whole region	4202	BaseHarv	2034 - 2050	1.8	17.3	24.6	2.9	-1	1.3 (0.8, 1.9)	41.9 (35.2, 46.3)
	Whole region	4202	LowHarv	2017 - 2025	2.9	25	26.6	2.4	-2.2	-0.5 (-0.8, 0)	51.6 (46.7, 55.2)
	Whole region	4202	LowHarv	2034 - 2050	2.4	23.6	25.2	1.8	-1.6	-0.3 (-0.6, 0.1)	48.8 (44.5, 51.6)
	Whole region	4202	MaxHarv	2017 - 2025	2.8	24.4	26.5	3.1	-2.1	0.1 (-0.2, 0.6)	50.9 (46.1, 54.3)
	Whole region	4202	MaxHarv	2034 - 2050	1.6	12.5	24	3.1	-0.6	1.8 (1.3, 2.1)	36.6 (31.4, 40.4)
08 Kymenlaakso	Currently protected	70	NoHarv	2017 - 2025	0	0.5	0.6	0	-0.1	-0.1 (-0.1, 0)	1.1 (1, 1.2)
	Currently protected	70	NoHarv	2034 - 2050	0	0.7	0.6	0	0	0 (0, 0)	1.3 (1.3, 1.4)

	10% target	351	NoHarv	2017 - 2025	0.3	2.7	3.4	0	-0.3	-0.3 (-0.3, -0.3)	6.1 (5.7, 6.4)
	10% target	351	NoHarv	2034 - 2050	0.2	3.5	3.4	0	-0.2	-0.2 (-0.2, -0.2)	6.9 (6.5, 7.1)
	Whole region	3273	BaseHarv	2017 - 2025	2.1	18.1	27.5	2.3	-1.4	0.4 (0.1, 0.7)	45.5 (41.4, 49.5)
	Whole region	3273	BaseHarv	2034 - 2050	1.1	9	25.5	2.1	-0.3	1.3 (0.8, 1.6)	34.5 (30.1, 38.9)
	Whole region	3273	LowHarv	2017 - 2025	2.1	18.3	27.5	1.9	-1.4	0 (-0.3, 0.3)	45.7 (41.7, 49.6)
	Whole region	3273	LowHarv	2034 - 2050	1.7	16.1	26.4	1.5	-0.9	0.2 (-0.1, 0.6)	42.4 (38.3, 45.3)
	Whole region	3273	MaxHarv	2017 - 2025	2.1	17.7	27.4	2.5	-1.3	0.6 (0.3, 0.9)	45.1 (41, 48.9)
	Whole region	3273	MaxHarv	2034 - 2050	1.1	6.9	25	1.7	-0.3	1.1 (0.4, 1.5)	31.9 (29.2, 34.9)
09 South Karelia	Currently protected	43	NoHarv	2017 - 2025	0	0.3	0.4	0	0	0 (0, 0)	0.7 (0.6, 0.7)
	Currently protected	43	NoHarv	2034 - 2050	0	0.4	0.4	0	0	0 (0, 0)	0.8 (0.8, 0.8)
	10% target	442	NoHarv	2017 - 2025	0.3	3.3	2.6	0	-0.4	-0.4 (-0.4, -0.3)	5.9 (5.5, 6.3)
	10% target	442	NoHarv	2034 - 2050	0.2	4.3	2.6	0	-0.3	-0.3 (-0.3, -0.2)	6.9 (6.5, 7.2)
	Whole region	4147	BaseHarv	2017 - 2025	2.5	21.9	40.2	2.9	-1.7	0.5 (0.2, 0.9)	62.1 (56.3, 66.4)
	Whole region	4147	BaseHarv	2034 - 2050	1.4	10.8	37.6	2.6	-0.3	1.7 (1.1, 2)	48.4 (43.1, 53.9)
	Whole region	4147	LowHarv	2017 - 2025	2.5	22.2	40.2	2.4	-1.7	0.1 (-0.3, 0.4)	62.4 (56.7, 66.6)
	Whole region	4147	LowHarv	2034 - 2050	2	19.8	38.7	1.8	-1.1	0.2 (-0.1, 0.7)	58.6 (53.8, 61.9)
	Whole region	4147	MaxHarv	2017 - 2025	2.5	21.4	40.1	3.1	-1.6	0.8 (0.5, 1.2)	61.5 (55.8, 65.8)
	Whole region	4147	MaxHarv	2034 - 2050	1.3	8.4	37	2.2	-0.3	1.4 (0.7, 1.9)	45.4 (42.1, 48.4)
10 South Savo	Currently protected	327	NoHarv	2017 - 2025	0.2	2.5	3.3	0	-0.2	-0.2 (-0.3, -0.2)	5.7 (5.3, 6.1)
	Currently protected	327	NoHarv	2034 - 2050	0.2	3.2	3.2	0	-0.2	-0.2 (-0.2, -0.1)	6.5 (6.2, 6.8)
	10% target	1140	NoHarv	2017 - 2025	0.8	8.8	9.3	0	-0.9	-0.9 (-1, -0.8)	18.1 (16.6, 19)
	10% target	1140	NoHarv	2034 - 2050	0.6	11.4	9.3	0	-0.7	-0.7 (-0.8, -0.5)	20.7 (19.4, 21.5)
	Whole region	10733	BaseHarv	2017 - 2025	6.3	58.3	95.5	7.6	-4	1.7 (0.8, 2.8)	153.7 (137.8, 167)
	Whole region	10733	BaseHarv	2034 - 2050	3	25.2	88.7	5.9	-0.2	4.4 (2.7, 5.5)	113.9 (100.9, 128)
	Whole region	10733	LowHarv	2017 - 2025	6.3	59.2	95.4	6.2	-4.2	0.5 (-0.5, 1.6)	154.6 (139.2, 167.3)
	Whole region	10733	LowHarv	2034 - 2050	4.8	49.3	90.9	4.8	-2.2	1.3 (0.4, 2.5)	140.2 (126.8, 151.1)
	Whole region	10733	MaxHarv	2017 - 2025	6.3	57.1	95.2	8.3	-3.8	2.4 (1.6, 3.5)	152.2 (136.6, 165.2)
	Whole region	10733	MaxHarv	2034 - 2050	2.9	19.9	87.1	4.8	-0.2	3.5 (1.6, 5.1)	107 (99.3, 116.1)
11 North Savo	Currently protected	242	NoHarv	2017 - 2025	0.2	1.7	1.1	0	-0.2	-0.2 (-0.2, -0.2)	2.9 (2.6, 3.1)
	Currently protected	242	NoHarv	2034 - 2050	0.1	2.3	1.1	0	-0.1	-0.1 (-0.2, -0.1)	3.4 (3.2, 3.6)
	10% target	1409	NoHarv	2017 - 2025	1	10.9	6	0	-1.1	-1.1 (-1.2, -1)	16.8 (15.3, 17.9)
	10% target	1409	NoHarv	2034 - 2050	0.8	13.8	6	0	-0.8	-0.8 (-0.9, -0.7)	19.7 (18.2, 20.5)
	Whole region	13808	BaseHarv	2017 - 2025	8.8	75	161.7	7.7	-6.1	-0.5 (-1.7, 1)	236.6 (223.1, 247.6)
	Whole region	13808	BaseHarv	2034 - 2050	5.7	58.1	155.8	7.9	-2.4	3.6 (2.1, 5.6)	213.8 (194.9, 226.8)
	Whole region	13808	LowHarv	2017 - 2025	8.8	75.7	161.5	6.3	-6.2	-1.5 (-2.7, -0.1)	237.3 (223.9, 248.4)
	Whole region	13808	LowHarv	2034 - 2050	7	75.7	157.3	4.8	-4.2	-0.8 (-1.8, 0.5)	233 (220.6, 242.8)
	Whole region	13808	MaxHarv	2017 - 2025	8.8	73.8	161.3	8.4	-5.9	0.3 (-0.9, 1.7)	235.1 (221.9, 246)

	Whole region	13808	MaxHarv	2034 - 2050	4.9	43.6	154	9.1	-1.1	5.9 (4.6, 7.3)	197.5 (180.5, 211.4)
12 North Karelia	Currently protected	443	NoHarv	2017 - 2025	0.3	3	4.3	0	-0.3	-0.3 (-0.3, -0.2)	7.3 (6.7, 7.7)
	Currently protected	443	NoHarv	2034 - 2050	0.2	4.1	4.2	0	-0.2	-0.2 (-0.3, -0.2)	8.3 (7.9, 8.6)
	10% target	1460	NoHarv	2017 - 2025	1	10.4	17.3	0	-1.1	-1.1 (-1.2, -1)	27.6 (26.1, 28.9)
	10% target	1460	NoHarv	2034 - 2050	0.8	13.5	17.2	0	-0.8	-0.8 (-0.9, -0.7)	30.8 (29.5, 31.8)
	Whole region	15335	BaseHarv	2017 - 2025	8.9	79.9	231.9	6.6	-6.5	-1.7 (-2.9, -0.4)	311.8 (293.8, 327.5)
	Whole region	15335	BaseHarv	2034 - 2050	6.7	71.7	226.5	6.8	-3.2	1.7 (0.7, 3)	298.2 (284, 311.6)
	Whole region	15335	LowHarv	2017 - 2025	9	81.2	231.9	5.4	-6.8	-2.9 (-4.2, -1.5)	313.1 (295, 328.6)
	Whole region	15335	LowHarv	2034 - 2050	7.2	89.8	228.5	4.1	-4.9	-2 (-3.3, -0.7)	318.4 (304.2, 332.2)
	Whole region	15335	MaxHarv	2017 - 2025	8.9	78.9	231.5	7.2	-6.1	-1 (-2.2, 0.4)	310.5 (292.5, 325.9)
	Whole region	15335	MaxHarv	2034 - 2050	6.3	61.6	224.9	8.1	-2.4	3.6 (2.4, 5)	286.5 (271.8, 300.4)
13 Central Finland	Currently protected	244	NoHarv	2017 - 2025	0.2	1.7	2.3	0	-0.2	-0.2 (-0.2, -0.2)	4 (3.7, 4.3)
	Currently protected	244	NoHarv	2034 - 2050	0.1	2.3	2.3	0	-0.1	-0.1 (-0.2, -0.1)	4.6 (4.4, 4.8)
	10% target	1364	NoHarv	2017 - 2025	0.9	10.4	8.4	0	-1.1	-1.1 (-1.2, -1)	18.8 (17.3, 20)
	10% target	1364	NoHarv	2034 - 2050	0.8	13.5	8.3	0	-0.8	-0.8 (-0.9, -0.7)	21.8 (20.6, 22.7)
	Whole region	13423	BaseHarv	2017 - 2025	8.2	72.9	139.4	7.6	-6.5	-0.8 (-2, 0.6)	212.2 (196, 224.9)
	Whole region	13423	BaseHarv	2034 - 2050	4.9	53.8	132.2	7.9	-2.2	3.9 (1.8, 6.2)	186 (162.3, 201.9)
	Whole region	13423	LowHarv	2017 - 2025	8.2	73.5	139.3	6.3	-6.5	-1.8 (-3, -0.6)	212.8 (197, 225.5)
	Whole region	13423	LowHarv	2034 - 2050	6.5	74.1	133.7	4.8	-4.2	-0.8 (-1.8, 0.5)	207.9 (194.2, 217.8)
	Whole region	13423	MaxHarv	2017 - 2025	8.2	71.7	139.1	8.3	-6.3	-0.1 (-1.2, 1.2)	210.8 (194.7, 223.3)
	Whole region	13423	MaxHarv	2034 - 2050	4.2	39.4	130.1	8.7	-1	5.8 (4.3, 7.2)	169.5 (150.7, 186.3)
14 South Ostrobothnia	Currently protected	134	NoHarv	2017 - 2025	0.1	0.7	1	0	-0.1	-0.1 (-0.1, -0.1)	1.7 (1.6, 1.9)
	Currently protected	134	NoHarv	2034 - 2050	0.1	1.1	1	0	-0.1	-0.1 (-0.1, -0.1)	2.1 (2, 2.2)
	10% target	946	NoHarv	2017 - 2025	0.6	6.1	4.9	0	-0.7	-0.7 (-0.8, -0.6)	11 (10, 11.8)
	10% target	946	NoHarv	2034 - 2050	0.5	8.6	4.8	0	-0.5	-0.5 (-0.6, -0.4)	13.4 (12.7, 14.1)
	Whole region	9330	BaseHarv	2017 - 2025	5.3	44.7	151.8	3.8	-4.7	-1.9 (-2.8, -1)	196.5 (184.9, 207.7)
	Whole region	9330	BaseHarv	2034 - 2050	3.9	42.8	148.4	3.9	-2.4	0.5 (-0.3, 1.5)	191.2 (178.9, 199.5)
	Whole region	9330	LowHarv	2017 - 2025	5.4	45.2	151.8	3.1	-4.8	-2.5 (-3.4, -1.6)	196.9 (185.2, 208.2)
	Whole region	9330	LowHarv	2034 - 2050	4.3	53.1	149.5	2.3	-3.5	-1.8 (-2.6, -1)	202.7 (192.4, 211.1)
	Whole region	9330	MaxHarv	2017 - 2025	5.3	44.2	151.6	4.1	-4.6	-1.6 (-2.4, -0.6)	195.7 (184.1, 206.9)
	Whole region	9330	MaxHarv	2034 - 2050	3.6	36.5	147.3	4.7	-1.9	1.6 (0.8, 3.1)	183.8 (170.1, 192.2)
15 Ostrobothnia	Currently protected	196	NoHarv	2017 - 2025	0.1	1	1.6	0	-0.2	-0.2 (-0.2, -0.1)	2.6 (2.5, 2.8)
	Currently protected	196	NoHarv	2034 - 2050	0.1	1.6	1.6	0	-0.1	-0.1 (-0.1, -0.1)	3.2 (3.1, 3.3)
	10% target	482	NoHarv	2017 - 2025	0.3	3	2.6	0	-0.4	-0.4 (-0.4, -0.3)	5.5 (5.2, 5.8)
	10% target	482	NoHarv	2034 - 2050	0.3	4.3	2.5	0	-0.3	-0.3 (-0.3, -0.3)	6.8 (6.4, 7.1)
	Whole region	5204	BaseHarv	2017 - 2025	3.2	26.2	56.7	2.8	-2.9	-0.9 (-1.3, -0.3)	82.9 (78.5, 87.4)
	Whole region	5204	BaseHarv	2034 - 2050	2.4	24.1	54.9	2.9	-1.6	0.5 (0.1, 1.1)	79 (74.4, 82.9)

	Whole region	5204	LowHarv	2017 - 2025	3.2	26.5	56.6	2.3	-2.9	-1.3 (-1.8, -0.8)	83.1 (78.7, 87.6)
	Whole region	5204	LowHarv	2034 - 2050	2.6	29.9	55.5	1.7	-2.1	-0.9 (-1.3, -0.4)	85.4 (81.2, 89)
	Whole region	5204	MaxHarv	2017 - 2025	3.2	25.8	56.6	3	-2.8	-0.6 (-1.1, -0.1)	82.4 (78, 86.9)
	Whole region	5204	MaxHarv	2034 - 2050	2.1	20.1	54.3	3.4	-1.3	1.3 (0.8, 2.1)	74.4 (68.9, 78.7)
16 Central Ostrobothnia	Currently protected	120	NoHarv	2017 - 2025	0.1	0.6	1.3	0	-0.1	-0.1 (-0.1, -0.1)	1.9 (1.7, 2)
	Currently protected	120	NoHarv	2034 - 2050	0.1	1	1.3	0	-0.1	-0.1 (-0.1, -0.1)	2.2 (2.1, 2.3)
	10% target	256	NoHarv	2017 - 2025	0.2	1.5	1.8	0	-0.2	-0.2 (-0.2, -0.1)	3.3 (3, 3.5)
	10% target	256	NoHarv	2034 - 2050	0.1	2.1	1.8	0	-0.1	-0.1 (-0.1, -0.1)	3.9 (3.8, 4.1)
	Whole region	3442	BaseHarv	2017 - 2025	1.9	16	68.9	1.2	-1.5	-0.6 (-0.9, -0.2)	84.9 (79.6, 89.6)
	Whole region	3442	BaseHarv	2034 - 2050	1.4	16	67.7	1.3	-0.7	0.3 (0, 0.6)	83.7 (77.6, 88.3)
	Whole region	3442	LowHarv	2017 - 2025	1.9	16.2	68.9	1	-1.5	-0.8 (-1.1, -0.4)	85 (79.7, 89.7)
	Whole region	3442	LowHarv	2034 - 2050	1.5	19.4	68.2	0.8	-1.1	-0.5 (-0.8, -0.2)	87.6 (82.3, 91.9)
	Whole region	3442	MaxHarv	2017 - 2025	1.9	15.8	68.8	1.3	-1.4	-0.5 (-0.8, -0.1)	84.7 (79.4, 89.3)
	Whole region	3442	MaxHarv	2034 - 2050	1.3	13.9	67.4	1.5	-0.5	0.6 (0.3, 1)	81.3 (75.4, 85.8)
17 North Ostrobothnia	Currently protected	1032	NoHarv	2017 - 2025	0.5	4.5	6.4	0	-0.6	-0.6 (-0.7, -0.5)	11 (10.1, 11.7)
	Currently protected	1032	NoHarv	2034 - 2050	0.5	7.2	6.4	0	-0.5	-0.5 (-0.6, -0.4)	13.6 (12.7, 14.3)
	10% target	2121	NoHarv	2017 - 2025	1.1	10.3	14.4	0	-1.2	-1.2 (-1.4, -1)	24.7 (23, 26.2)
	10% target	2121	NoHarv	2034 - 2050	1	15.7	14.4	0	-1	-1 (-1.1, -0.9)	30 (28.1, 31.3)
	Whole region	25655	BaseHarv	2017 - 2025	12.6	100.8	534.9	7.3	-9.3	-4 (-5.9, -1.4)	635.7 (603.6, 670)
	Whole region	25655	BaseHarv	2034 - 2050	9.8	109.4	528.4	7.5	-4.9	0.5 (-1.6, 2.7)	637.9 (606.6, 673.1)
	Whole region	25655	LowHarv	2017 - 2025	12.6	102.1	534.9	6.1	-9.8	-5.4 (-7.5, -2.8)	637 (604.5, 671.5)
	Whole region	25655	LowHarv	2034 - 2050	10.6	131.3	531.2	4.5	-7.4	-4.2 (-6.3, -2.1)	662.6 (630.4, 697.3)
	Whole region	25655	MaxHarv	2017 - 2025	12.5	99.7	534.5	8	-8.9	-3.1 (-5.1, -0.5)	634.3 (602.4, 668.5)
	Whole region	25655	MaxHarv	2034 - 2050	9.2	95.7	526.5	9	-3.7	2.9 (0.7, 5.5)	622.1 (591, 657)
18 Kainuu	Currently protected	815	NoHarv	2017 - 2025	0.4	4.5	4.8	0	-0.5	-0.5 (-0.6, -0.4)	9.3 (8.5, 9.9)
	Currently protected	815	NoHarv	2034 - 2050	0.4	6.5	4.8	0	-0.4	-0.4 (-0.5, -0.3)	11.3 (10.5, 11.9)
	10% target	1356	NoHarv	2017 - 2025	0.7	7.5	6.8	0	-0.8	-0.8 (-0.9, -0.7)	14.3 (13.2, 15.3)
	10% target	1356	NoHarv	2034 - 2050	0.6	10.9	6.8	0	-0.7	-0.7 (-0.8, -0.6)	17.6 (16.8, 18.5)
	Whole region	16235	BaseHarv	2017 - 2025	8.3	66.2	300.3	4	-6.8	-4 (-5.2, -2.5)	366.5 (346.4, 385.5)
	Whole region	16235	BaseHarv	2034 - 2050	6.7	77.5	296.4	4.1	-4.3	-1.3 (-2.5, 0)	373.9 (355.2, 393.8)
	Whole region	16235	LowHarv	2017 - 2025	8.3	67	300.4	3.3	-7.1	-4.7 (-6, -3.2)	367.4 (347.3, 386.4)
	Whole region	16235	LowHarv	2034 - 2050	7.1	90.5	298.7	2.5	-5.7	-4 (-5.2, -2.7)	389.1 (370, 409.8)
	Whole region	16235	MaxHarv	2017 - 2025	8.3	65.6	300.1	4.3	-6.6	-3.5 (-4.8, -2)	365.7 (345.6, 384.7)
	Whole region	16235	MaxHarv	2034 - 2050	6.4	70	295.3	4.9	-3.6	-0.1 (-1.3, 1.2)	365.4 (347.2, 385)
19 Lapland	Currently protected	11800	NoHarv	2017 - 2025	2	26.2	141.1	0	-3	-3 (-3.6, -2.3)	167.3 (157.6, 176.7)
	Currently protected	11800	NoHarv	2034 - 2050	2.4	39.6	141.8	0	-3.3	-3.3 (-4.1, -2.4)	181.4 (169.1, 194.3)
	10% target	11800	NoHarv	2017 - 2025	2	26.3	141.4	0	-3	-3 (-3.5, -2.4)	167.8 (158.1, 175)

	10% target	11800	NoHarv	2034 - 2050	2.4	40	142.1	0	-3.3	-3.3 (-3.9, -2.6)	182.1 (170.2, 191.9)
	Whole region	55307	BaseHarv	2017 - 2025	16.1	146.6	533	5.2	-16.7	-13 (-16.3, -9.2)	679.5 (636.9, 723.5)
	Whole region	55307	BaseHarv	2034 - 2050	15.1	200.5	524.8	5.5	-13.7	-9.8 (-12.9, -6.2)	725.3 (671.5, 775.4)
	Whole region	55307	LowHarv	2017 - 2025	16.2	147.7	533.2	4.3	-17.1	-14 (-17.2, -10.3)	681 (638.2, 725)
	Whole region	55307	LowHarv	2034 - 2050	15.9	218.9	531.9	3.3	-16.2	-13.9 (-17.1, -10.2)	750.8 (696.2, 800.7)
	Whole region	55307	MaxHarv	2017 - 2025	16.1	145.9	532.4	5.7	-16.6	-12.5 (-15.8, -8.6)	678.3 (635.8, 722.3)
	Whole region	55307	MaxHarv	2034 - 2050	14.5	189.4	521.3	6.5	-12.5	-7.8 (-10.8, -4.2)	710.7 (657.1, 759.9)

Table S3_3. Area (km²), forest harvesting scenario, period, volume growth (Mm³ a⁻¹), C storages in trees+ground vegetation and soils (TgC), harvested volume (Mm³ a⁻¹), NEE (TgCO₂eq a⁻¹), climate regulation service net C sequestration (NBE) (TgCO₂eq a⁻¹), and climate regulation service C storage (TgC) for forested areas of the 18 administrative regions of mainland Finland (see Fig. 2 in main paper), for two levels of protected areas (currently protected areas, assuming an increase to 10% strictly protected area), and time periods. PREBAS model simulations have been made assuming the RCP4.5 climate change scenario. Results for forests are shown as averages for several years to reduce the annual variability caused by changes in climate conditions. Negative NBE values indicate sinks and positive values sources. Uncertainty estimates (95%) are shown for the NBE and C storage values. Results are shown separately for the protected areas and the whole forested area (protected + managed area) of Finland. For the protected areas, the NoHarv scenario is assumed. For the whole area, the results of three harvesting intensity scenarios (BaseHarv, LowHarv and MaxHarv) for the forested areas outside the currently strictly protected areas are shown. These harvesting intensity scenarios indicate the implications of intensive vs. conservation-oriented forestry for the managed forest land with respect to key C variables. The climate regulation services net C sequestration (NBE) and C storage are calculated according to SEEA definitions. Corresponding results assuming current climate conditions are shown in Table S3_2. See main paper for details.

Region		Area km2	Scenario	Period	Volume growth $Mm^3 a^{-1}$	C storage above ground TgC	C storage soils TgC	Harv. volume $Mm^3 a^{-1}$	NEE $TgCO_2eq\ a^{-1}$	Climate serv. net C sequestration (NBE) (95%) $TgCO_2eq\ a^{-1}$	Climate serv. C storage (95%) TgC
01 Uusimaa	Currently protected	250	NoHarv	2017 - 2025	0.2	1.8	1.5	0	-0.2	-0.2 (-0.2, -0.2)	3.2 (2.9, 3.4)
	Currently protected	250	NoHarv	2034 - 2050	0.2	2.6	1.5	0	-0.2	-0.2 (-0.2, -0.2)	4.1 (3.8, 4.3)
	10% target	680	NoHarv	2017 - 2025	0.5	4.9	3	0	-0.6	-0.6 (-0.7, -0.5)	8 (7.3, 8.6)
	10% target	680	NoHarv	2034 - 2050	0.5	7.2	3.1	0	-0.6	-0.6 (-0.6, -0.5)	10.3 (9.6, 10.8)
	Whole region	5761	BaseHarv	2017 - 2025	4.1	32.4	30.4	4	-3.7	-0.8 (-1.4, 0)	62.7 (56.3, 67.7)
	Whole region	5761	BaseHarv	2034 - 2050	3.2	27.9	28.8	4.2	-2.6	0.7 (-0.2, 1.7)	56.7 (45, 63.2)
	Whole region	5761	LowHarv	2017 - 2025	4.2	32.7	30.3	3.3	-3.8	-1.4 (-2, -0.6)	63 (56.8, 67.9)
	Whole region	5761	LowHarv	2034 - 2050	4	37.1	29.5	2.5	-3.6	-1.8 (-2.4, -1.1)	66.6 (59.8, 70.8)
	Whole region	5761	MaxHarv	2017 - 2025	4.1	31.8	30.3	4.4	-3.7	-0.4 (-1.1, 0.3)	62.1 (55.7, 67)
	Whole region	5761	MaxHarv	2034 - 2050	2.8	21	28.3	4.8	-2	1.7 (1, 2.4)	49.2 (40.9, 56.6)
02 Southwest Finland	Currently protected	216	NoHarv	2017 - 2025	0.2	1.3	3.4	0	-0.2	-0.2 (-0.2, -0.1)	4.7 (4.5, 4.9)
	Currently protected	216	NoHarv	2034 - 2050	0.2	2.1	3.4	0	-0.2	-0.2 (-0.2, -0.1)	5.5 (5.3, 5.8)
	10% target	754	NoHarv	2017 - 2025	0.6	4.9	8.9	0	-0.6	-0.6 (-0.7, -0.5)	13.8 (13, 14.6)
	10% target	754	NoHarv	2034 - 2050	0.6	7.6	8.9	0	-0.6	-0.6 (-0.7, -0.5)	16.5 (15.4, 17.3)
	Whole region	6394	BaseHarv	2017 - 2025	4.4	33.7	37.4	3.6	-4.2	-1.6 (-2.2, -0.7)	71.1 (65.5, 76.1)
	Whole region	6394	BaseHarv	2034 - 2050	3.9	34.9	36.3	3.7	-3.4	-0.7 (-1.4, 0.2)	71.2 (64, 76)
	Whole region	6394	LowHarv	2017 - 2025	4.5	34	37.4	2.9	-4.2	-2.1 (-2.8, -1.3)	71.4 (65.8, 76.4)
	Whole region	6394	LowHarv	2034 - 2050	4.4	43.1	37.2	2.2	-4.4	-2.8 (-3.6, -1.8)	80.3 (74.6, 84.9)
	Whole region	6394	MaxHarv	2017 - 2025	4.4	33.2	37.3	3.9	-4.1	-1.3 (-1.9, -0.5)	70.5 (64.9, 75.5)
	Whole region	6394	MaxHarv	2034 - 2050	3.7	29.6	35.7	4.4	-3	0.3 (-0.4, 1.2)	65.3 (57.2, 69.9)
04 Satakunta	Currently protected	120	NoHarv	2017 - 2025	0.1	0.7	1	0	-0.1	-0.1 (-0.1, -0.1)	1.7 (1.6, 1.8)
	Currently protected	120	NoHarv	2034 - 2050	0.1	1.2	1	0	-0.1	-0.1 (-0.1, -0.1)	2.2 (2, 2.3)
	10% target	541	NoHarv	2017 - 2025	0.4	3.8	2.9	0	-0.5	-0.5 (-0.5, -0.4)	6.7 (6.2, 7.2)
	10% target	541	NoHarv	2034 - 2050	0.4	5.6	2.9	0	-0.4	-0.4 (-0.5, -0.3)	8.5 (8, 8.9)

	Whole region	5294	BaseHarv	2017 - 2025	3.8	28.9	57.4	3.1	-3	-0.8 (-1.3, -0.1)	86.3 (81.2, 90.6)
	Whole region	5294	BaseHarv	2034 - 2050	3.3	27.1	55.8	3.2	-2.2	0.1 (-0.5, 0.8)	82.9 (76.9, 86.8)
	Whole region	5294	LowHarv	2017 - 2025	3.8	29.2	57.4	2.5	-3.1	-1.2 (-1.7, -0.5)	86.6 (81.3, 90.9)
	Whole region	5294	LowHarv	2034 - 2050	3.7	34.8	56.6	1.9	-3.1	-1.8 (-2.4, -1)	91.4 (86.9, 95.7)
	Whole region	5294	MaxHarv	2017 - 2025	3.8	28.4	57.2	3.4	-2.9	-0.5 (-1, 0.2)	85.7 (80.6, 89.9)
	Whole region	5294	MaxHarv	2034 - 2050	3.1	22.3	55.2	3.8	-1.9	1 (0.3, 1.7)	77.5 (70.8, 81.8)
05 Kanta-Häme	Currently protected	86	NoHarv	2017 - 2025	0.1	0.7	0.5	0	-0.1	-0.1 (-0.1, -0.1)	1.1 (1, 1.2)
	Currently protected	86	NoHarv	2034 - 2050	0.1	1	0.5	0	-0.1	-0.1 (-0.1, -0.1)	1.4 (1.3, 1.5)
	10% target	394	NoHarv	2017 - 2025	0.3	3.3	2.3	0	-0.3	-0.3 (-0.4, -0.3)	5.5 (5.1, 5.9)
	10% target	394	NoHarv	2034 - 2050	0.3	4.4	2.3	0	-0.3	-0.3 (-0.3, -0.3)	6.7 (6.2, 7)
	Whole region	3554	BaseHarv	2017 - 2025	2.6	20.6	31.4	2.7	-1.8	0.2 (-0.1, 0.6)	52 (47.1, 55.8)
	Whole region	3554	BaseHarv	2034 - 2050	2	14.6	29.7	2.7	-1.1	1 (0.6, 1.4)	44.4 (37.4, 48.5)
	Whole region	3554	LowHarv	2017 - 2025	2.6	20.8	31.4	2.2	-1.8	-0.2 (-0.5, 0.3)	52.2 (47.3, 55.8)
	Whole region	3554	LowHarv	2034 - 2050	2.6	21.3	30.5	1.7	-1.9	-0.7 (-1.1, -0.2)	51.8 (47.3, 55.1)
	Whole region	3554	MaxHarv	2017 - 2025	2.6	20.2	31.3	2.9	-1.7	0.5 (0.1, 0.9)	51.5 (46.6, 55.2)
	Whole region	3554	MaxHarv	2034 - 2050	1.8	10.5	29.2	2.7	-0.9	1.2 (0.5, 1.6)	39.7 (35.5, 43.6)
06 Pirkanmaa	Currently protected	215	NoHarv	2017 - 2025	0.2	1.7	1.2	0	-0.2	-0.2 (-0.2, -0.2)	2.9 (2.6, 3.2)
	Currently protected	215	NoHarv	2034 - 2050	0.2	2.4	1.3	0	-0.2	-0.2 (-0.2, -0.1)	3.6 (3.4, 3.8)
	10% target	1077	NoHarv	2017 - 2025	0.9	8.8	5.4	0	-0.9	-0.9 (-1.1, -0.8)	14.2 (12.8, 15.2)
	10% target	1077	NoHarv	2034 - 2050	0.8	11.9	5.4	0	-0.8	-0.8 (-0.9, -0.7)	17.3 (16.1, 18.1)
	Whole region	10031	BaseHarv	2017 - 2025	7.3	59.5	92.1	6.3	-5.7	-1.1 (-2.3, 0.2)	151.6 (140.2, 161)
	Whole region	10031	BaseHarv	2034 - 2050	6	53.4	88.4	6.5	-4.1	0.8 (-0.4, 2.4)	141.8 (126.8, 151.8)
	Whole region	10031	LowHarv	2017 - 2025	7.3	59.9	92	5.2	-5.7	-2 (-3.1, -0.7)	151.9 (141, 161.5)
	Whole region	10031	LowHarv	2034 - 2050	7	67.5	89.8	3.9	-5.7	-2.9 (-4, -1.7)	157.3 (146.9, 166.4)
	Whole region	10031	MaxHarv	2017 - 2025	7.2	58.5	91.8	6.8	-5.6	-0.6 (-1.6, 0.7)	150.3 (139.2, 159.8)
	Whole region	10031	MaxHarv	2034 - 2050	5.5	43.1	87.2	7.7	-3.3	2.6 (1.2, 4.1)	130.3 (113.2, 141.6)
07 Päijät-Häme	Currently protected	76	NoHarv	2017 - 2025	0.1	0.6	1.1	0	-0.1	-0.1 (-0.1, -0.1)	1.7 (1.6, 1.8)
	Currently protected	76	NoHarv	2034 - 2050	0.1	0.8	1.1	0	-0.1	-0.1 (-0.1, 0)	2 (1.9, 2)
	10% target	480	NoHarv	2017 - 2025	0.4	4	4.3	0	-0.4	-0.4 (-0.5, -0.4)	8.3 (7.8, 8.7)
	10% target	480	NoHarv	2034 - 2050	0.3	5.3	4.3	0	-0.4	-0.4 (-0.4, -0.3)	9.6 (9.1, 10)
	Whole region	4202	BaseHarv	2017 - 2025	3.1	25.1	26.6	2.9	-2.3	-0.2 (-0.7, 0.3)	51.7 (46.8, 55.3)
	Whole region	4202	BaseHarv	2034 - 2050	2.5	20.7	25.1	2.9	-1.7	0.6 (0.1, 1.2)	45.8 (37.4, 49.7)
	Whole region	4202	LowHarv	2017 - 2025	3.1	25.2	26.6	2.4	-2.3	-0.6 (-1.1, 0)	51.8 (46.9, 55.6)
	Whole region	4202	LowHarv	2034 - 2050	3	27	25.7	1.8	-2.4	-1.1 (-1.5, -0.6)	52.7 (47.5, 55.8)
	Whole region	4202	MaxHarv	2017 - 2025	3.1	24.6	26.5	3.1	-2.2	0 (-0.4, 0.6)	51.1 (46.3, 54.7)
	Whole region	4202	MaxHarv	2034 - 2050	2.3	15.9	24.5	3.5	-1.3	1.3 (0.9, 1.8)	40.5 (33.6, 44.8)
08 Kymenlaakso	Currently protected	70	NoHarv	2017 - 2025	0.1	0.5	0.6	0	-0.1	-0.1 (-0.1, 0)	1.1 (1, 1.2)

	Currently protected	70	NoHarv	2034 - 2050	0.1	0.7	0.6	0	-0.1	-0.1 (-0.1, 0)	1.4 (1.3, 1.4)
	10% target	351	NoHarv	2017 - 2025	0.3	2.7	3.4	0	-0.3	-0.3 (-0.4, -0.3)	6.1 (5.6, 6.4)
	10% target	351	NoHarv	2034 - 2050	0.3	3.8	3.4	0	-0.3	-0.3 (-0.3, -0.2)	7.2 (6.7, 7.5)
	Whole region	3273	BaseHarv	2017 - 2025	2.3	18.2	27.5	2.3	-1.5	0.3 (-0.1, 0.6)	45.6 (41.2, 49.6)
	Whole region	3273	BaseHarv	2034 - 2050	1.6	11.4	26	2.4	-0.8	1 (0.6, 1.4)	37.4 (31.3, 42.5)
	Whole region	3273	LowHarv	2017 - 2025	2.3	18.4	27.4	1.9	-1.5	-0.1 (-0.5, 0.3)	45.8 (41.6, 49.8)
	Whole region	3273	LowHarv	2034 - 2050	2.2	18.8	26.9	1.5	-1.6	-0.5 (-0.9, -0.1)	45.7 (40.8, 49.2)
	Whole region	3273	MaxHarv	2017 - 2025	2.2	17.8	27.3	2.5	-1.4	0.5 (0.1, 0.8)	45.1 (40.8, 49.1)
	Whole region	3273	MaxHarv	2034 - 2050	1.5	8.3	25.5	2.1	-0.7	0.9 (0.2, 1.5)	33.8 (30.7, 37.8)
09 South Karelia	Currently protected	43	NoHarv	2017 - 2025	0	0.3	0.4	0	0	0 (0, 0)	0.7 (0.6, 0.7)
	Currently protected	43	NoHarv	2034 - 2050	0	0.5	0.4	0	0	0 (0, 0)	0.8 (0.8, 0.9)
	10% target	442	NoHarv	2017 - 2025	0.3	3.3	2.6	0	-0.4	-0.4 (-0.4, -0.3)	6 (5.5, 6.4)
	10% target	442	NoHarv	2034 - 2050	0.3	4.7	2.6	0	-0.3	-0.3 (-0.4, -0.3)	7.3 (6.8, 7.7)
	Whole region	4147	BaseHarv	2017 - 2025	2.7	22.1	40.4	2.9	-1.8	0.4 (-0.1, 0.8)	62.4 (57.2, 67.1)
	Whole region	4147	BaseHarv	2034 - 2050	2	14	38.4	2.9	-1	1.3 (0.8, 1.8)	52.4 (45.3, 59.4)
	Whole region	4147	LowHarv	2017 - 2025	2.7	22.4	40.3	2.4	-1.9	-0.1 (-0.6, 0.4)	62.7 (57.6, 67.1)
	Whole region	4147	LowHarv	2034 - 2050	2.8	23.3	39.6	1.8	-2	-0.7 (-1.1, -0.1)	62.9 (57.4, 67.3)
	Whole region	4147	MaxHarv	2017 - 2025	2.7	21.6	40.2	3.1	-1.7	0.7 (0.2, 1.2)	61.9 (56.7, 66.4)
	Whole region	4147	MaxHarv	2034 - 2050	1.8	10.2	37.8	2.7	-0.8	1.2 (0.4, 1.8)	48 (43.8, 52.7)
10 South Savo	Currently protected	327	NoHarv	2017 - 2025	0.2	2.5	3.3	0	-0.3	-0.3 (-0.3, -0.2)	5.8 (5.2, 6.2)
	Currently protected	327	NoHarv	2034 - 2050	0.2	3.5	3.3	0	-0.2	-0.2 (-0.3, -0.2)	6.8 (6.4, 7.1)
	10% target	1140	NoHarv	2017 - 2025	0.8	8.9	9.4	0	-0.9	-0.9 (-1.1, -0.8)	18.2 (17.1, 19.3)
	10% target	1140	NoHarv	2034 - 2050	0.8	12.4	9.4	0	-0.9	-0.9 (-0.9, -0.7)	21.8 (20.7, 22.6)
	Whole region	10733	BaseHarv	2017 - 2025	6.9	58.9	95.6	7.6	-4.4	1.3 (0.3, 2.4)	154.6 (138.8, 167.9)
	Whole region	10733	BaseHarv	2034 - 2050	4.4	32.8	90.4	7.1	-1.8	3.7 (2.2, 4.8)	123.2 (105.6, 140.2)
	Whole region	10733	LowHarv	2017 - 2025	7	59.8	95.6	6.2	-4.6	0.1 (-1, 1.2)	155.4 (140.1, 168)
	Whole region	10733	LowHarv	2034 - 2050	6.7	58.1	93.2	4.8	-4.4	-1 (-2.1, 0.6)	151.3 (134.1, 161.6)
	Whole region	10733	MaxHarv	2017 - 2025	6.9	57.7	95.3	8.3	-4.1	2.1 (1, 3.2)	153.1 (137.5, 166)
	Whole region	10733	MaxHarv	2034 - 2050	4.1	24.4	89.1	5.9	-1.6	3 (0.9, 4.6)	113.5 (103.2, 125.1)
11 North Savo	Currently protected	242	NoHarv	2017 - 2025	0.2	1.8	1.1	0	-0.2	-0.2 (-0.2, -0.2)	2.9 (2.6, 3.1)
	Currently protected	242	NoHarv	2034 - 2050	0.2	2.5	1.1	0	-0.2	-0.2 (-0.2, -0.2)	3.7 (3.5, 3.8)
	10% target	1409	NoHarv	2017 - 2025	1.1	11	6	0	-1.2	-1.2 (-1.4, -1)	16.9 (15.4, 18.3)
	10% target	1409	NoHarv	2034 - 2050	1	15	6	0	-1	-1 (-1.2, -0.9)	21 (19.6, 22.1)
	Whole region	13808	BaseHarv	2017 - 2025	9.7	76.4	161.3	7.7	-6.8	-1.1 (-2.6, 0.4)	237.8 (225.2, 251.2)
	Whole region	13808	BaseHarv	2034 - 2050	7.9	68.6	156.8	7.9	-4.6	1.4 (-0.3, 3.4)	225.4 (207.2, 240.8)
	Whole region	13808	LowHarv	2017 - 2025	9.7	77.1	161.2	6.3	-6.9	-2.2 (-3.8, -0.5)	238.3 (226, 251.8)
	Whole region	13808	LowHarv	2034 - 2050	9.3	87.5	158.6	4.8	-6.9	-3.5 (-5.1, -1.7)	246.1 (233.4, 259.8)

	Whole region	13808	MaxHarv	2017 - 2025	9.6	75.2	161	8.4	-6.5	-0.4 (-1.9, 1.3)	236.2 (223.8, 249.5)
	Whole region	13808	MaxHarv	2034 - 2050	7.2	55.2	155.3	9.4	-3.5	3.8 (1.7, 5.7)	210.5 (190.7, 228.1)
12 North Karelia	Currently protected	443	NoHarv	2017 - 2025	0.3	3.1	4.3	0	-0.3	-0.3 (-0.4, -0.3)	7.4 (6.8, 7.8)
	Currently protected	443	NoHarv	2034 - 2050	0.3	4.5	4.3	0	-0.3	-0.3 (-0.4, -0.3)	8.8 (8.4, 9.2)
	10% target	1460	NoHarv	2017 - 2025	1.1	10.5	17.3	0	-1.2	-1.2 (-1.4, -1)	27.8 (26.5, 29.1)
	10% target	1460	NoHarv	2034 - 2050	1	14.9	17.3	0	-1.1	-1.1 (-1.2, -0.9)	32.3 (30.8, 33.5)
	Whole region	15335	BaseHarv	2017 - 2025	9.9	81.9	232.2	6.6	-7.4	-2.6 (-4.3, -1.1)	314.1 (295.2, 330)
	Whole region	15335	BaseHarv	2034 - 2050	9.1	83.8	228.6	6.8	-5.9	-1 (-2.9, 1.1)	312.4 (294.4, 329.1)
	Whole region	15335	LowHarv	2017 - 2025	10	82.9	232.2	5.4	-7.7	-3.8 (-5.5, -2.2)	315.1 (296.1, 331)
	Whole region	15335	LowHarv	2034 - 2050	10	103.6	230.8	4.1	-8.2	-5.3 (-6.9, -3.4)	334.4 (316, 351)
	Whole region	15335	MaxHarv	2017 - 2025	9.9	80.8	231.8	7.2	-7.1	-1.9 (-3.6, -0.3)	312.6 (293.9, 328.6)
	Whole region	15335	MaxHarv	2034 - 2050	8.8	72.5	227.4	8.1	-5	0.8 (-1.2, 3)	300 (281.2, 317.3)
13 Central Finland	Currently protected	244	NoHarv	2017 - 2025	0.2	1.8	2.3	0	-0.2	-0.2 (-0.2, -0.2)	4.1 (3.8, 4.3)
	Currently protected	244	NoHarv	2034 - 2050	0.2	2.6	2.3	0	-0.2	-0.2 (-0.2, -0.2)	4.9 (4.6, 5.1)
	10% target	1364	NoHarv	2017 - 2025	1	10.6	8.4	0	-1.2	-1.2 (-1.3, -1)	18.9 (17.5, 20.1)
	10% target	1364	NoHarv	2034 - 2050	1	14.7	8.4	0	-1	-1 (-1.2, -0.9)	23.2 (21.8, 24)
	Whole region	13423	BaseHarv	2017 - 2025	9.2	74.5	140	7.6	-7.2	-1.5 (-3, 0)	214.5 (200.4, 227.9)
	Whole region	13423	BaseHarv	2034 - 2050	7.1	66.4	134.4	8	-4.6	1.4 (-0.6, 3.9)	200.8 (175.9, 216.7)
	Whole region	13423	LowHarv	2017 - 2025	9.2	75.1	140	6.3	-7.2	-2.6 (-4, -1)	215.1 (201.3, 228.5)
	Whole region	13423	LowHarv	2034 - 2050	8.9	86.2	136.4	4.8	-7	-3.6 (-5.1, -1.9)	222.6 (208.9, 233.6)
	Whole region	13423	MaxHarv	2017 - 2025	9.1	73.3	139.7	8.3	-7	-0.8 (-2.2, 0.7)	213 (199.1, 226.3)
	Whole region	13423	MaxHarv	2034 - 2050	6.3	51.9	132.6	9.4	-3.4	3.9 (1.6, 5.8)	184.4 (159.7, 202.5)
14 South Ostrobothnia	Currently protected	134	NoHarv	2017 - 2025	0.1	0.7	1	0	-0.1	-0.1 (-0.1, -0.1)	1.8 (1.6, 1.9)
	Currently protected	134	NoHarv	2034 - 2050	0.1	1.2	1	0	-0.1	-0.1 (-0.1, -0.1)	2.3 (2.1, 2.4)
	10% target	946	NoHarv	2017 - 2025	0.7	6.2	4.9	0	-0.8	-0.8 (-0.9, -0.7)	11.1 (10.1, 12)
	10% target	946	NoHarv	2034 - 2050	0.7	9.6	4.9	0	-0.7	-0.7 (-0.8, -0.6)	14.5 (13.7, 15.2)
	Whole region	9330	BaseHarv	2017 - 2025	6.1	45.9	152.2	3.8	-5.4	-2.6 (-3.7, -1.6)	198.2 (188.5, 209.9)
	Whole region	9330	BaseHarv	2034 - 2050	5.6	51.3	149.7	3.9	-4.3	-1.5 (-2.7, -0.2)	201 (190.3, 211.4)
	Whole region	9330	LowHarv	2017 - 2025	6.1	46.3	152.2	3.1	-5.5	-3.2 (-4.3, -2.2)	198.6 (188.8, 210.4)
	Whole region	9330	LowHarv	2034 - 2050	6.2	62.3	151.3	2.3	-5.7	-4 (-5.2, -3.1)	213.6 (203.7, 223.9)
	Whole region	9330	MaxHarv	2017 - 2025	6.1	45.4	152	4.1	-5.3	-2.3 (-3.3, -1.2)	197.4 (187.7, 209.1)
	Whole region	9330	MaxHarv	2034 - 2050	5.4	44.7	149	4.7	-3.8	-0.3 (-1.6, 1.3)	193.6 (182.1, 204)
15 Ostrobothnia	Currently protected	196	NoHarv	2017 - 2025	0.1	1.1	1.6	0	-0.2	-0.2 (-0.2, -0.1)	2.6 (2.4, 2.8)
	Currently protected	196	NoHarv	2034 - 2050	0.2	1.8	1.6	0	-0.2	-0.2 (-0.2, -0.1)	3.4 (3.2, 3.5)
	10% target	482	NoHarv	2017 - 2025	0.4	3	2.6	0	-0.4	-0.4 (-0.5, -0.4)	5.6 (5.2, 5.9)
	10% target	482	NoHarv	2034 - 2050	0.4	4.7	2.6	0	-0.4	-0.4 (-0.4, -0.3)	7.3 (6.8, 7.6)
	Whole region	5204	BaseHarv	2017 - 2025	3.6	26.9	56.5	2.8	-3.2	-1.1 (-1.6, -0.5)	83.4 (77.9, 87.8)

	Whole region	5204	BaseHarv	2034 - 2050	3.2	27.7	55.4	2.9	-2.5	-0.4 (-1.1, 0.3)	83.1 (78, 87.3)
	Whole region	5204	LowHarv	2017 - 2025	3.6	27.2	56.5	2.3	-3.2	-1.5 (-2, -0.9)	83.6 (78.1, 88)
	Whole region	5204	LowHarv	2034 - 2050	3.6	34.4	56	1.7	-3.2	-2 (-2.6, -1.4)	90.4 (85.6, 94.3)
	Whole region	5204	MaxHarv	2017 - 2025	3.6	26.5	56.4	3	-3.1	-0.9 (-1.3, -0.3)	82.9 (77.4, 87.3)
	Whole region	5204	MaxHarv	2034 - 2050	3.1	23.7	54.9	3.4	-2.2	0.4 (-0.3, 1.3)	78.6 (73, 83)
16 Central Ostrobothnia	Currently protected	120	NoHarv	2017 - 2025	0.1	0.6	1.3	0	-0.1	-0.1 (-0.1, -0.1)	1.9 (1.8, 2)
	Currently protected	120	NoHarv	2034 - 2050	0.1	1.1	1.3	0	-0.1	-0.1 (-0.1, -0.1)	2.4 (2.2, 2.5)
	10% target	256	NoHarv	2017 - 2025	0.2	1.5	1.8	0	-0.2	-0.2 (-0.2, -0.2)	3.3 (3, 3.5)
	10% target	256	NoHarv	2034 - 2050	0.2	2.5	1.8	0	-0.2	-0.2 (-0.2, -0.2)	4.3 (4.1, 4.5)
	Whole region	3442	BaseHarv	2017 - 2025	2.3	16.6	68.6	1.2	-1.8	-0.9 (-1.3, -0.6)	85.2 (79.7, 89.4)
	Whole region	3442	BaseHarv	2034 - 2050	2.1	19.7	67.8	1.3	-1.5	-0.6 (-1.1, -0.1)	87.5 (81.7, 92.3)
	Whole region	3442	LowHarv	2017 - 2025	2.3	16.8	68.6	1	-1.9	-1.2 (-1.5, -0.8)	85.3 (79.9, 89.6)
	Whole region	3442	LowHarv	2034 - 2050	2.3	23.4	68.3	0.8	-1.9	-1.4 (-1.9, -1)	91.8 (86, 96.6)
	Whole region	3442	MaxHarv	2017 - 2025	2.2	16.4	68.5	1.3	-1.8	-0.8 (-1.2, -0.4)	84.9 (79.5, 89.2)
	Whole region	3442	MaxHarv	2034 - 2050	2	17.6	67.5	1.5	-1.3	-0.2 (-0.7, 0.3)	85.1 (79.2, 89.9)
17 North Ostrobothnia	Currently protected	1032	NoHarv	2017 - 2025	0.6	4.7	6.5	0	-0.7	-0.7 (-0.8, -0.6)	11.2 (10.3, 12)
	Currently protected	1032	NoHarv	2034 - 2050	0.7	8.3	6.6	0	-0.8	-0.8 (-0.9, -0.7)	14.9 (14, 15.9)
	10% target	2121	NoHarv	2017 - 2025	1.3	10.6	14.6	0	-1.5	-1.5 (-1.8, -1.2)	25.2 (23.3, 26.8)
	10% target	2121	NoHarv	2034 - 2050	1.4	18.1	14.7	0	-1.6	-1.6 (-1.9, -1.3)	32.8 (31.2, 34.5)
	Whole region	25655	BaseHarv	2017 - 2025	15.1	105.8	536.4	7.3	-12.1	-6.8 (-10.2, -3.7)	642.2 (605.6, 679.5)
	Whole region	25655	BaseHarv	2034 - 2050	14.8	136.2	532.6	7.5	-10.9	-5.4 (-9.4, -1.9)	668.8 (625.3, 708.4)
	Whole region	25655	LowHarv	2017 - 2025	15.1	106.9	536.4	6.1	-12.6	-8.2 (-11.9, -5.2)	643.3 (606.9, 680.7)
	Whole region	25655	LowHarv	2034 - 2050	16.2	159.7	536	4.5	-14.1	-10.8 (-14.7, -7.4)	695.7 (652.9, 734.8)
	Whole region	25655	MaxHarv	2017 - 2025	15	104.7	536	8	-11.8	-5.9 (-9.4, -2.9)	640.7 (604.4, 678)
	Whole region	25655	MaxHarv	2034 - 2050	14.2	121.8	530.9	9.1	-9.6	-3 (-7.2, 0.9)	652.7 (609.9, 691.9)
18 Kainuu	Currently protected	815	NoHarv	2017 - 2025	0.5	4.6	4.8	0	-0.6	-0.6 (-0.7, -0.5)	9.4 (8.6, 10.1)
	Currently protected	815	NoHarv	2034 - 2050	0.5	7.4	4.9	0	-0.6	-0.6 (-0.7, -0.5)	12.2 (11.5, 13)
	10% target	1356	NoHarv	2017 - 2025	0.9	7.7	6.9	0	-1	-1 (-1.2, -0.8)	14.6 (13.4, 15.7)
	10% target	1356	NoHarv	2034 - 2050	0.9	12.4	6.9	0	-1	-1 (-1.2, -0.8)	19.3 (18.3, 20.6)
	Whole region	16235	BaseHarv	2017 - 2025	9.8	69.5	300	4	-8.6	-5.7 (-7.8, -3.9)	369.5 (351.2, 388.1)
	Whole region	16235	BaseHarv	2034 - 2050	9.8	94.2	298.2	4.1	-8.1	-5.1 (-7.5, -2.9)	392.4 (371, 414.9)
	Whole region	16235	LowHarv	2017 - 2025	9.8	70.2	300	3.3	-8.8	-6.5 (-8.6, -4.7)	370.2 (351.9, 388.9)
	Whole region	16235	LowHarv	2034 - 2050	10.6	107.8	300.1	2.5	-9.8	-8 (-10.3, -5.8)	407.9 (386.5, 429.3)
	Whole region	16235	MaxHarv	2017 - 2025	9.7	68.9	299.8	4.3	-8.4	-5.2 (-7.4, -3.4)	368.7 (350.4, 387.3)
	Whole region	16235	MaxHarv	2034 - 2050	9.5	86.4	297.3	4.9	-7.4	-3.8 (-6.3, -1.7)	383.7 (362.6, 406.1)
19 Lapland	Currently protected	11800	NoHarv	2017 - 2025	2.6	26.8	140.5	0	-3.6	-3.6 (-4.7, -2.7)	167.2 (156.5, 176.4)
	Currently protected	11800	NoHarv	2034 - 2050	4.3	49.8	141.7	0	-5.9	-5.9 (-7.5, -4.5)	191.5 (177.3, 204.4)

	10% target	11800	NoHarv	2017 - 2025	2.7	27	141	0	-3.6	-3.6 (-4.5, -2.7)	168 (158.2, 176.8)
	10% target	11800	NoHarv	2034 - 2050	4.3	50	142.2	0	-5.9	-5.9 (-7.6, -4.6)	192.2 (178.9, 205.7)
	Whole region	55307	BaseHarv	2017 - 2025	20	153	531.1	5.2	-21	-17.3 (-23.3, -12.3)	684.1 (638.5, 721)
	Whole region	55307	BaseHarv	2034 - 2050	25.6	253.7	530.4	5.5	-27.2	-23.3 (-30.8, -15.8)	784 (725.8, 839.2)
	Whole region	55307	LowHarv	2017 - 2025	20.1	154	531.5	4.3	-21.4	-18.3 (-24.2, -13.4)	685.6 (639.7, 723)
	Whole region	55307	LowHarv	2034 - 2050	26.9	273.3	536.5	3.3	-30.2	-27.8 (-35.2, -20.3)	809.7 (752, 864.6)
	Whole region	55307	MaxHarv	2017 - 2025	19.9	152.2	530.6	5.7	-20.8	-16.7 (-22.7, -11.8)	682.8 (637.3, 719.8)
	Whole region	55307	MaxHarv	2034 - 2050	24.8	241.7	527.6	6.6	-25.9	-21.2 (-28.7, -13.8)	769.3 (711.7, 824.9)

Table S_4. Estimated anthropogenic emissions ($TgCO_2eq\ a^{-1}$) in the 18 administrative regions of mainland Finland (see Fig. 2 in main paper) in the current situation (year 2019), for the WEM scenario in years 2030 and 2050, and for WAM scenario in 2030. The WAM scenario for 2050 was not estimated because of uncertainties in spatial allocation of emissions (implementation of CCS/BECCS measures after 2040). See main paper for details.

	Scenario			
**Region**	**Current**	**WEM 2030**	**WEM 2050**	**WAM 2030**
Uusimaa	13,502	8,765	5,813	7,411
Southwest Finland	3,820	2,708	1,894	2,318
Satakunta	2,813	1,572	0,937	1,242
Kanta-Häme	1,559	1,389	0,725	1,064
Pirkanmaa	3,214	2,135	1,259	1,849
Päijät-Häme	1,466	1,162	0,661	0,961
Kymenlaakso	1,565	1,345	0,784	1,151
South Karelia	1,843	1,602	1,115	1,440
South Savo	1,278	0,923	0,525	0,741
North Savo	2,316	1,727	1,078	1,462
North Karelia	1,373	1,102	0,609	0,877
Central Finland	1,933	1,170	0,663	0,977
South Ostrobothnia	2,703	1,791	1,250	1,470
Ostrobothnia	2,146	1,362	0,872	1,062
Central Ostrobothnia	0,901	0,610	0,458	0,520
North Ostrobothnia	8,050	6,723	2,249	6,264
Kainuu	0,719	0,465	0,288	0,402
Lapland	2,893	2,452	1,761	2,214
